# Effectiveness and Safety of Apixaban in over 3.9 Million People with Atrial Fibrillation: A Systematic Review and Meta-Analysis

**DOI:** 10.3390/jcm11133788

**Published:** 2022-06-30

**Authors:** Benjamin J. R. Buckley, Deirdre A. Lane, Peter Calvert, Juqian Zhang, David Gent, C. Daniel Mullins, Paul Dorian, Shun Kohsaka, Stefan H. Hohnloser, Gregory Y. H. Lip

**Affiliations:** 1 Liverpool Centre for Cardiovascular Science, University of Liverpool and Liverpool Heart & Chest Hospital, Liverpool L14 3PE, UK; deirdre.lane@liverpool.ac.uk (D.A.L.); pcalvert@doctors.org.uk (P.C.); juqian.zhang@liverpool.ac.uk (J.Z.); david.gent@nhs.net (D.G.); gregory.lip@liverpool.ac.uk (G.Y.H.L.); 2 Cardiovascular and Metabolic Medicine, Institute of Life Course and Medical Sciences, Faculty of Health and Life Sciences, University of Liverpool, Liverpool L69 3BX, UK; 3 Department of Clinical Medicine, Aalborg University, P.O. Box 159, DK-9100 Aalborg, Denmark; 4 PHSR Department, School of Pharmacy, University of Maryland, Baltimore, MD 20742, USA; daniel.mullins@rx.umaryland.edu; 5 Division of Cardiology, St Michael’s Hospital, University of Toronto, Toronto, ON M5S 1A1, Canada; paul.dorian@unityhealth.to; 6 Department of Cardiology, Keio University, Tokyo 108-8345, Japan; sk@keio.jp; 7 Department of Cardiology, J.W. Goethe University, 60590 Frankfurt, Germany; hohnloser@em.uni-frankfurt.de; 8 Centre of Thrombosis and Hemostasis, University of Mainz, 55122 Mainz, Germany

**Keywords:** apixaban, atrial fibrillation, stroke, major bleeding, anticoagulant, secondary prevention

## Abstract

Background: There is a plethora of real-world data on the safety and effectiveness of direct-acting oral anticoagulants (DOACs); however, study heterogeneity has contributed to inconsistent findings. We compared the effectiveness and safety of apixaban with those of other direct-acting oral anticoagulants (DOACs) and vitamin K antagonists (VKA e.g., warfarin). Methods: A systematic review and meta-analysis was conducted retrieving data from PubMed, SCOPUS and Web of Science from January 2009 to December 2021. Studies that evaluated apixaban (intervention) prescribed for adults (aged 18 years or older) with AF for stroke prevention compared to other DOACs or VKAs were identified. Primary outcomes included stroke/systemic embolism (SE), all-cause mortality, and major bleeding. Secondary outcomes were intracranial haemorrhage (ICH) and ischaemic stroke. Randomised controlled trials and non-randomised trials were considered for inclusion. Results: In total, 67 studies were included, and 38 studies were meta-analysed. Participants taking apixaban had significantly lower stroke/SE compared to patients taking VKAs (relative risk (RR) 0.77, 95% confidence interval (CI) 0.64–0.93, I^2^ = 94%) and dabigatran (RR 0.84, 95% CI 0.74–0.95, I^2^ = 66%), but not to patients administered rivaroxaban. There was no statistical difference in mortality between apixaban and VKAs or apixaban and dabigatran. Compared to patients administered rivaroxaban, participants taking apixaban had lower mortality rates (RR 0.83, 95% CI 0.71–0.96, I^2^ = 96%). Apixaban was associated with a significantly lower risk of major bleeding compared to VKAs (RR 0.58, 95% CI 0.52–0.65, I^2^ = 90%), dabigatran (RR 0.79, 95% CI 0.70–0.88, I^2^ = 78%) and rivaroxaban (RR 0.61, 95% CI 0.53–0.70, I^2^ = 87%). Conclusions: Apixaban was associated with a better overall safety and effectiveness profile compared to VKAs and other DOACs.

## 1. Introduction

Anticoagulation is the fundamental priority for the prevention of stroke in people diagnosed with atrial fibrillation (AF) and is one of the pillars of guideline-recommended AF management [1,2]. The efficacy of direct-acting oral anticoagulants (DOACs) versus warfarin has received considerable attention in the last decade. DOACs have generally [3,4], although not always [5], demonstrated favourable outcomes for stroke and mortality. Typically, DOACs are now favoured over warfarin for stroke prevention in AF due to their superior safety profile regarding intracranial haemorrhage (all DOACs) and major bleeding (some DOACs) [1,6]. However, agreement on the most favourable DOAC in terms of effectiveness and safety is challenging, particularly given that there are no head-to-head comparison trials.

There is a plethora of real-world data on the safety, and to a lesser extent, the effectiveness of DOACs, but the disparity in study populations, inclusion and exclusion criteria, and statistical analyses, has resulted in inconsistent findings and several unanswered questions. Further, our understanding of the potential impact of geographical region, study design and age on such outcomes is lacking. Addressing these gaps in the current evidence base will allow clinicians and patients to make better, evidence-informed decisions when selecting a DOAC to prevent stroke in people with AF.

Given apixaban is the most commonly used DOAC [7], the aims of this systematic review and meta-analysis were to compare the effectiveness and safety of apixaban with those of other DOACs and vitamin K antagonists (VKA e.g., warfarin). First, we sought to determine if apixaban was more effective (reduced stroke and mortality) and safer (reduced major bleeding) than dabigatran, rivaroxaban, edoxaban, and VKAs for patients with AF. Second, we investigated how geographical region (Asia, Europe, North America) and age (≥75/<75 years of age) may impact the effectiveness and safety of apixaban.

## 2. Methods

This systematic review was registered in the International Prospective Register of Systematic Reviews—PROSPERO (CRD42021236826) and conducted in accordance with the Preferred Reporting Items for Systematic Reviews and Meta-analyses (PRISMA) guidelines [8].

### 2.1. Study Inclusion Criteria

We included studies carried out in any setting that evaluated apixaban (intervention) prescribed for adults (aged 18 years or older) with AF for stroke prevention compared to other DOACs or VKAs (e.g., warfarin). Primary outcomes included stroke or any thromboembolic event (stroke/SE composite), all-cause mortality, and major bleeding. Secondary outcomes included intracranial haemorrhage (ICH) and ischaemic stroke. Definitions used for each outcome were employed by the primary trials and may not be consistent between studies. All randomised controlled trials and non-randomised studies, pre–post studies and interrupted time series were considered for inclusion. Cross-sectional studies, case reports and qualitative studies were excluded.

### 2.2. Search Strategy

The search strategy was developed by the review team who selected all key terms. Medical subject headings (MesH) terms and synonyms for the different terms such as “atrial fibrillation”, “apixaban” and “stroke” were used and combined with Boolean operators, proximity operators, truncations and wildcards. PubMed, SCOPUS and Web of Science were searched from 1 January 2009 to 21 December 2021 for relevant studies (refer to Supplement S1 for full search strategies). Database searches were initiated from 2009 rather than inception, because the first DOAC trial (RE-LY) was published in 2009 [9]. There were no language restrictions; however, availability of the full text was a requirement for inclusion. Search results were managed using EndNote X9.3.3.

### 2.3. Study Selection

Two reviewers (B.J.R.B., J.Z.), independently and in duplicate, screened the titles and abstracts of the studies retrieved by the databases against the search criteria. The full texts of all potentially relevant articles were retrieved and independently assessed by the reviewers (B.J.R.B., P.C., D.A.L., M.C., D.G.). Any disagreement was resolved through discussion with the first author (B.J.R.B.).

### 2.4. Data Extraction

Data extraction was conducted independently by five reviewers (B.J.R.B., P.C., D.A.L., M.C., D.G.), with at least 20% checked by (B.J.R.B.) to ensure consistency/accuracy. The following information was extracted: (i) authors, year, country, reference; (ii) study design with inclusion/exclusion criteria; (iii) study aim; (iv) intervention and comparator characteristics (*n* = age, sex, CHA_2_DS_2_-VASc, HAS-BLED); (v) outcomes (effectiveness and safety); (vi) follow-up time points; (vii) results; (viii) study conclusions; (ix) risk of bias assessment.

### 2.5. Risk of Bias Assessment

Five authors (B.J.R.B., P.C., D.A.L., M.C., D.G.) independently assessed the individual studies for risk of bias in duplicate, and any discrepancies were resolved via discussion or referral to a third reviewer, as required. The Cochrane Risk of Bias v.2 (RoB2) tool [10] was used to assess the risk of bias for randomised controlled trials (RCTs). The Risk Of Bias In Non-randomised Studies—of Interventions (ROBINS-I) [11] was used to assess the risk of bias for non-randomised studies.

### 2.6. Data Synthesis

Meta-analyses were conducted for comparable studies. Primary and secondary outcome effect measures with 95% confidence intervals were pooled using RevMan software [12]. Results are presented visually using Forest plots. For studies where quantitative data were too few or too heterogeneous, a narrative synthesis approach was used. Effect measures for dichotomous outcomes were analysed using the number of events and total sample size as reported in the included studies. Results of the selected studies were combined using the Mantel–Haenszel method. Effect sizes were expressed as relative risk and 95% confidence intervals. Heterogeneity was quantitatively assessed using Higgins’s index (I^2^), with 25%, 50% and 75% considered moderate, substantial and considerable heterogeneity, respectively. Random-effect models were applied allowing for between-study variability by weighting studies using a combination of intra- and inter-study variance.

### 2.7. Sub-Group and Sensitivity Analyses

Sub-group analyses were conducted (if sufficient data) to explore any impact of cohort age (≥75 and <75 years) and geographical region (North America, Asia, Europe) on the safety and effectiveness of apixaban compared to VKAs, dabigatran, and rivaroxaban. Sensitivity analyses were planned to explore the impact of studies deemed as ‘serious risk of bias’ on the safety and effectiveness of apixaban.

## 3. Results

**Figure 1:**
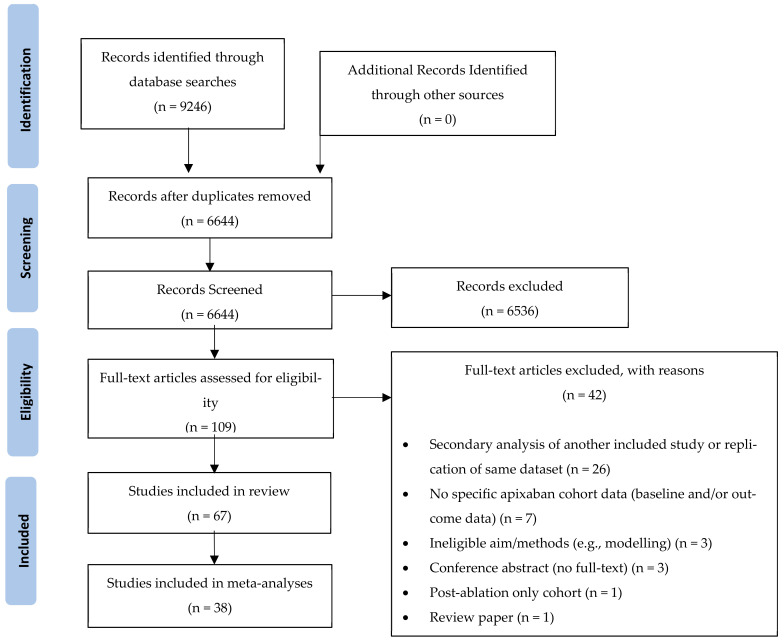
PRISMA Diagram depicting the screening and study selection process.

The database searches identified 9246 papers. After removal of duplicates, 6644 papers were included in the title and abstract screening, which resulted in 109 papers retrieved for full-text screening against the inclusion/exclusion criteria. Of these, 67 (61%) studies were included in the systematic review, and 38 (35%) studies were included in meta-analyses (Figure 1).

### 3.1. Characteristics of the Included Studies

The included studies were published between 2009 and 2021; two of them were randomised controlled trials [13,14], two were prospective cohort studies [15,16], and the remaining 63 were retrospective cohort studies (Supplementary File; Table S1). The sample size for the apixaban-treated cohorts ranged between *n* = 98 [17] and *n* = 353,897 patients [18]. The total sample of patients included in the review was 3,911,894, of which, 1,292,620 patients (33%) were taking apixaban. Mean/median patient age ranged from 62 [19] to 86 [20] years, and the proportion of females ranged between 15% [14] and 69% [21]. Of the 67 included studies, 34 were conducted in the U.S.A., 7 in Denmark, 3 each in Sweden, Spain, Norway, Japan, and Germany, 2 each in the UK and South Korea, 1 each in Taiwan, Canada, Thailand, France, and Singapore, 1 was international, with 39 participating countries, and 1 included data from Canada, the U.S.A. and the UK.

For the two included randomised controlled trials, one was deemed ‘low risk of bias’ [13], and one was deemed ‘some concerns’ [14], with the latter due to non-blinded participants. Overall, 64/65 real-world studies were deemed ‘moderate risk of bias‘, with one study deemed to be at serious risk of bias [22]. For all included real-world studies, the risk of bias was elevated due to confounding which was inherent in the study design. Further study-level detail regarding the risk of bias is reported within the Supplementary File, Table S2 and Figure S1.

### 3.2. Primary Outcomes

**Figure 2:**
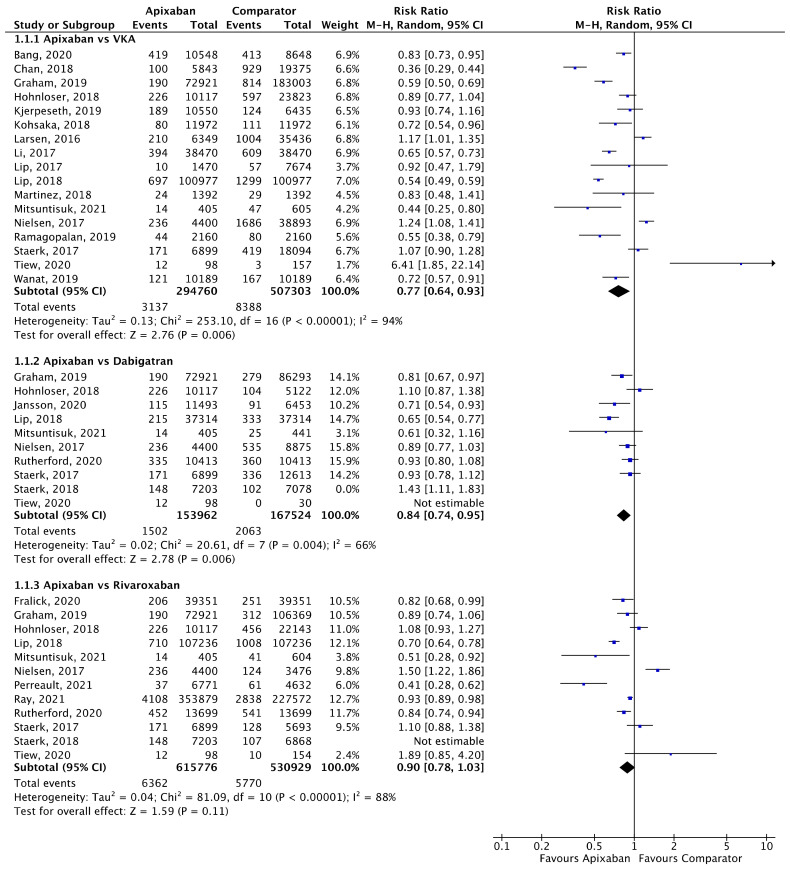
Comparison of apixaban to VKAs (1.1.1), dabigatran (1.1.2) and rivaroxaban (1.1.3) for stroke/SE [17,18,20,24,25,26,27,28,29,30,31,32,33,34,35,36,37,38,39,40,41,42,43].

**Figure 3:**
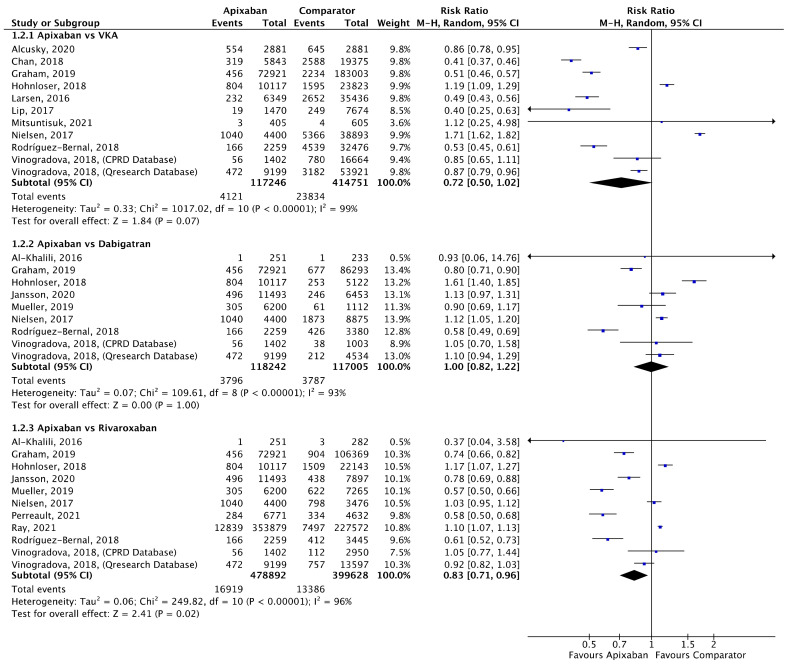
Comparison of apixaban to VKAs (1.2.1), dabigatran (1.2.2) and rivaroxaban (1.2.3) for mortality [16,18,21,25,26,27,30,32,34,35,39,44,45,46,47].

**Figure 4:**
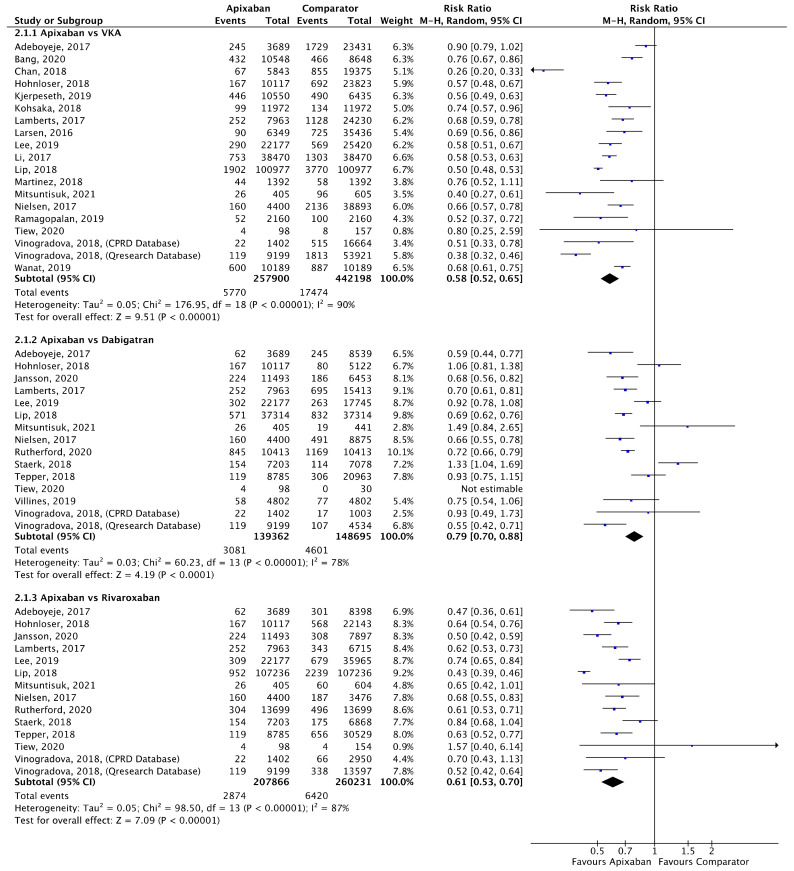
Comparison of apixaban to VKAs (2.1.1), dabigatran (2.1.2) and rivaroxaban (2.1.3) for major bleeding [16,17,20,23,24,25,27,28,29,30,31,33,34,35,36,38,39,40,41,48,49,50,51].

Meta-analyses for stroke/SE (Figure 2), mortality (Figure 3) and major bleeding (Figure 4) are presented below. Each analysis includes all eligible studies and compared apixaban with VKAs, dabigatran and rivaroxaban for each outcome. A comparison of apixaban with edoxaban was not possible due to only one eligible study including data for both drugs [23].

A total of 17 (*n* = 802,063), 10 (*n* = 321,486), and 12 (*n* = 1,146,705) studies compared apixaban to VKAs, dabigatran and rivaroxaban, respectively, and were included in meta-analyses investigating stroke/SE (Figure 2). Compared to VKAs, apixaban was associated with a significantly lower risk of stroke/SE (relative risk (RR) 0.77, 95% confidence interval (CI) 0.64–0.93, I^2^ = 94%). Compared to dabigatran, apixaban was associated with a significantly lower risk of stroke/SE (RR 0.84, 95% CI 0.74–0.95, I^2^ = 66%). There was no statistical difference in risk of stroke/SE between apixaban and rivaroxaban (RR 0.90, 95% CI 0.78–1.03, I^2^ = 88%).

A total of 10 (*n* = 533,997), 8 (*n* = 235,247) and 10 (*n* = 878,520) studies compared apixaban to VKAs, dabigatran and rivaroxaban, respectively, and were included in meta-analyses investigating mortality (Figure 3). There was no statistical difference in mortality between apixaban and VKAs (RR 0.72, 95% CI 0.50–1.00, I^2^ = 99%) or apixaban and dabigatran (RR 1.00, 95% CI 0.82–1.22, I^2^ = 93%). Compared to rivaroxaban, apixaban was associated with a significantly lower risk of mortality (RR 0.83, 95% CI 0.71–0.96, I^2^ = 96%).

A total of 18 (*n* = 700,098), 14 (*n* = 288,057) and 13 (*n* = 468,097) studies compared apixaban to VKAs, dabigatran and rivaroxaban, respectively, and were included in meta-analyses investigating major bleeding (Figure 4). Apixaban was associated with a significantly lower risk of major bleeding compared to VKAs (RR 0.58, 95% CI 0.52–0.65, I^2^ = 90%), dabigatran (RR 0.79, 95% CI 0.70–0.88, I^2^ = 78%) and rivaroxaban (RR 0.61, 95% CI 0.53–0.70, I^2^ = 87%).

### 3.3. Secondary Outcomes

**Figure 5:**
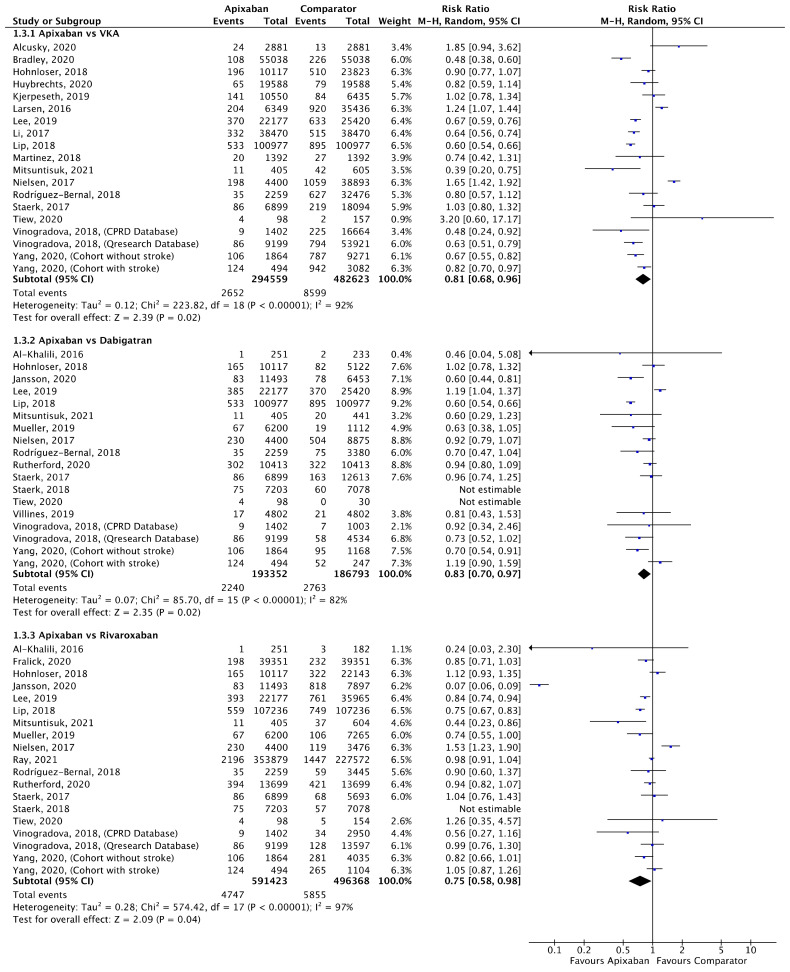
Comparison of apixaban to VKAs (1.3.1), dabigatran (1.3.2) and rivaroxaban (1.3.3) for ischaemic stroke [16,17,18,20,21,23,27,28,30,31,33,34,35,37,38,39,40,41,42,44,45,46,52,53,54].

**Figure 6:**
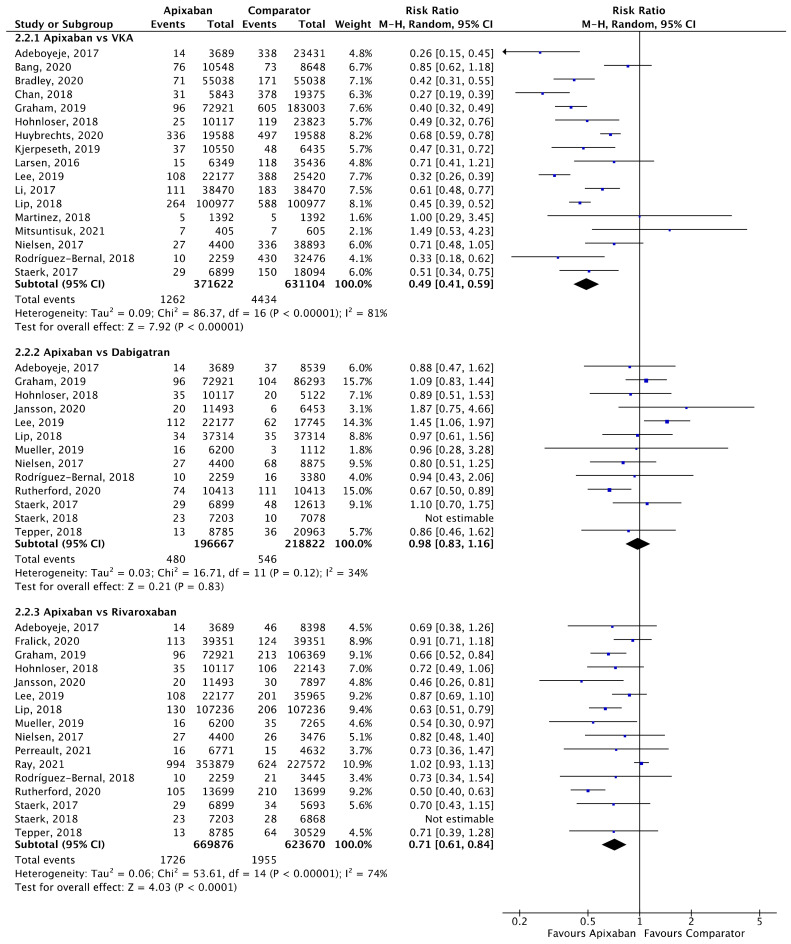
Comparison of apixaban to VKAs (2.2.1), dabigatran (2.2.2) and rivaroxaban (2.2.3) for ICH [18,20,23,24,25,26,27,28,30,31,33,34,35,37,39,40,41,42,44,46,47,48,50,52,53].

Meta-analyses for ischaemic stroke (Figure 5) and ICH (Figure 6) are presented below. Each analysis included all eligible studies and compared apixaban with VKAs, dabigatran and rivaroxaban for each outcome.

A total of 19 (*n* = 777,182), 16 (*n* = 380,145) and 17 (*n* = 1,087,791) studies compared apixaban to VKAs, dabigatran and rivaroxaban, respectively, and were included in meta-analyses investigating ischaemic stroke (Figure 5). Apixaban was associated with a significantly lower risk of ischaemic stroke compared to VKAs (RR 0.81, 95% CI 0.68–0.96, I^2^ = 92%), dabigatran (RR 0.83, 95% CI 0.70–0.97, I^2^ = 82%) and rivaroxaban (RR 0.75, 95% CI 0.58–0.98, I^2^ = 97%).

A total of 17 (*n* = 1,002,726), 13 (*n* = 415,489) and 16 (*n* = 1,293,546) studies compared apixaban to VKAs, dabigatran and rivaroxaban, respectively, and were included in meta-analyses investigating ICH (Figure 6). Apixaban was associated with a significantly lower rates of ICH compared to VKAs (RR 0.49, 95% CI 0.41–0.59, I^2^ = 81%) and rivaroxaban (RR 0.71, 95% CI 0.61–0.84, I^2^ = 74%), but not dabigatran (RR 0.98, 95% CI 0.83–1.16, I^2^ = 34%).

### 3.4. Sub-Group and Sensitivity Analyses

Sub-group meta-analyses exploring the impact of participants’ age (≥75 and <75 years) were not possible due to insufficient sub-group data stratified by consistent age boundaries within the primary trials. Sub-group analyses for the impact of geographic region (North America, Asia, Europe) on the safety and effectiveness of apixaban are presented in Figures S2–S4 (Supplement S3). Meta-analyses are presented for the primary outcomes stroke/SE, mortality and major bleeding comparing apixaban to VKAs, apixaban to dabigatran and apixaban to rivaroxaban stratified by geographic region.

### 3.5. Stratification by Geographic Region

Regarding the apixaban vs VKA comparisons, the relative risk of stroke/SE was significantly lower with apixaban in North America, but not in Asia or Europe. There was no difference in the relative risk of mortality across the geographic sub-groups, and the relative risk of major bleeding was lower with apixaban across all geographic sub-groups (Figure S2; Supplement S4). For the apixaban vs dabigatran comparisons, the relative risk of stroke/SE and mortality were significantly lower with apixaban in North America only. The relative risk of major bleeding was significantly lower with apixaban in North America and Europe (Figure S3; Supplement S4). For the apixaban vs rivaroxaban comparisons, the relative risk of stroke/SE was significantly lower with apixaban in North America only. The relative risk for mortality was significantly lower with apixaban in North America and Europe and could not be estimated in Asia (due to no eligible studies). The relative risk for major bleeding was significantly lower with apixaban across all geographic subgroups (Figure S4; Supplement S4).

Sensitivity analyses were not necessary to explore the impact of studies deemed ‘serious risk of bias’ on the safety and effectiveness of apixaban, as no studies with ‘serious risk of bias’ were included in the meta-analyses.

## 4. Discussion

Our systematic review and meta-analyses show that the use of apixaban was associated with improved effectiveness (reduced stroke/SE and ischaemic stroke) and safety profile (major bleeding and ICH) when compared with the use of VKAs. Compared with dabigatran, apixaban was associated with significantly lower stroke or systemic embolism, major bleeding events and ischaemic stroke but not mortality or ICH. Compared to rivaroxaban, apixaban was associated with significantly lower mortality, major bleeding, ischaemic stroke, and ICH but not stroke/SE. Some results varied when stratified by geographic region.

In the ARISTOTLE randomised controlled trial (*n* = 18,201 people with AF), apixaban was superior to warfarin for the prevention of stroke or systemic embolism [13]. A phase II randomised controlled trial, ARISTOTLE-J, showed that in Japanese patients with AF, apixaban was well tolerated, with lower rates of major bleeding than warfarin over 12 weeks. However, to determine the effectiveness and safety of apixaban with those of OACs other than VKAs (i.e., warfarin), namely, dabigatran, rivaroxaban and edoxaban, real-world studies are needed.

Our findings extend those of a previous systematic review and meta-analysis (16 studies with up to *n =* 266,598 people with AF included in the meta-analysis), which showed that the use of apixaban in cohort studies was associated with an overall similar effectiveness in reducing stroke and any thromboembolic events when compared with the use of warfarin (odds ratio 0.92, 95% CI 0.72–1.10) [4]. However, the previous review demonstrated a better safety profile for apixaban compared to warfarin, dabigatran, and rivaroxaban. A more recent systematic review and network meta-analysis (21 studies with 605,771 people with AF) [55] found that apixaban was associated with a lower risk of major bleeding compared to rivaroxaban (hazard ratio 1.8, 95% CI 1.6–2.1) and dabigatran (hazard ratio 1.4, 95% CI 1.2–1.6), which is in agreement with the findings of the present study. Menichelli et al. [55] did, however, showed a higher risk of stroke or systemic embolism with rivaroxaban compared to apixaban (hazard ratio 1.4, 95% CI 1.00–1.80) but not with dabigatran compared with apixaban, which is contrary to our findings, although their work included fewer studies and participants. The authors also did not find a mortality benefit for apixaban, whereas the present study found a mortality benefit for apixaban vs. rivaroxaban. Thus, the analysis of randomised controlled trials and cohort studies demonstrated differing (and not yet established) effectiveness and safety profiles in DOAC–DOAC comparisons.

A recent retrospective cohort study (published after the searches for this systematic review), including >580,000 US Medicare beneficiaries, found that rivaroxaban was associated with a higher adjusted risk for ischaemic or haemorrhagic events (hazard ratio 1.18 95% CI 1.12–1.24) compared to apixaban [18]. This present study was updated to include this work in the appropriate meta-analyses. The findings in the present review demonstrate an overall beneficial association for apixaban over rivaroxaban for stroke/SE, mortality, major bleeding, ischaemic stroke and ICH. These findings add to the body of evidence suggesting that apixaban is associated with a lower bleeding risk and greater thromboembolic protection compared with rivaroxaban. Further and more broadly, our findings provide evidence that, although apixaban was not associated with an improvement in all outcomes across all DOAC comparisons, none of the outcomes we investigated favoured either dabigatran or rivaroxaban when compared to apixaban.

### Limitations

The generally high heterogeneity in several meta-analyses makes it challenging to ascertain definitive conclusions. Furthermore, there was insufficient evidence from real-world studies to compare edoxaban with other commonly used DOACs. Other factors may have also influenced our findings, including inappropriate DOAC dosing and individual patient OAC adherence and associated comorbidities. Similarly, we did not investigate differences in DOAC dosage and associated study outcomes within this systematic review. Despite the reduced data on the number of participants receiving the standard or a lower dose apixaban, Proietti et al. previously demonstrated that the standard dose may be superior to a reduced dose of apixaban for the reduction of any thromboembolic event [4]. Indeed, OAC is only one aspect of holistic or integrated care management of AF [2], whereby adherence to such an approach and appropriate characterisation of AF patients have been associated with improved clinical outcomes [56,57]. Further, the addition of statin therapy to OAC has been shown to improve in-hospital prognosis of patients with acute ischaemic stroke [43] and reduce long-term major adverse cardiovascular events in patients with embolic stroke of undetermined source [58]. This is also important when considering the use of apixaban, given the need for twice-daily dosing compared to once-daily dosing for rivaroxaban, for example, and should be considered on a patient-by-patient and shared decision-making process. Finally, most real-world studies included in this review were of a retrospective cohort design, with innate and well-known limitations, although, adjusted effect measures or propensity score matched populations were used where possible.

## 5. Conclusions

In this systematic review and meta-analysis combining data from clinical trials and real-world studies with >3.9 million participants, apixaban was associated with a better overall safety and effectiveness profile compared to VKAs and other DOACs. Despite the use of random-effect models to estimate overall effect estimates, considerable heterogeneity was present in most meta-analyses, and this should be considered when interpreting the results.

## Data Availability

Not applicable as data is already published.

## Supplementary Materials

The following supporting information can be downloaded at: https://www.mdpi.com/article/10.3390/jcm11133788/s1, Supplement S1. Full search strategies. Supplement S2. Characteristics of the included studies. Supplement S3. Risk of bias of the included studies. Supplement S4. Sub-group meta-analyses. References [59,60,61,62,63,64,65,66,67,68,69,70,71,72,73,74,75,76,77,78,79,80,81,82,83,84] are cited in Supplementary Materials.
