# Supplementary material for: Effectiveness and Safety of Apixaban in over 3.9 Million People with Atrial Fibrillation: A Systematic Review and Meta-Analysis
Source: J Clin Med. 2022 Jun 30;11(13):3788. doi: 10.3390/jcm11133788 (PMC9267894; doi:10.3390/jcm11133788)
Supplement: Supplementary file 1 [file jcm-11-03788-s001.zip › jcm-1782349-supplementary.pdf]

Supplementary Material

Comparison of the effectiveness and safety of apixaban compared with oral anticoagulants: a systematic review and meta-analysis of real-world and trial evidence

Benjamin J. R. Buckley, Deirdre A. Lane, Peter Calvert, Juqian Zhang, David Gent, C. Daniel Mullins, Paul Dorian, Shun Kohsaka, Stefan H. Hohnloser, Gregory Y. H. Lip

Supplement S1. Full search strategies

Supplement S2. Characteristics of included studies

Supplement S3. Risk of bias of included studies

Supplement S4. Sub-group meta-analyses

Supplement S1. Full search strategies for Web of Science, Pubmed, and SCOPUS

Summary of hits
9246 Identified
2602 Duplicates removed
6644 Screened for full text review

Web of Science = 4,830
TS=(Atrial fibrillation)
AND
TS=(NOAC OR non vitamin k oral anticoagulant OR novel oral anticoagulant OR DOAC OR direct oral anticoagulant OR Apixaban OR Dabigatran OR Rivaroxaban OR Edoxaban OR vitamin K antagonist VKA OR warfarin OR coumadin OR 4hydroxycoumarin* OR acenocoumar* OR acenocumar* OR coumarin* OR cumarin* OR ethyl biscoumacetate OR phenprocoum* OR phenprocum* OR dicoumar* OR dicumar* OR fluindione OR phenindione OR clorindione OR diphenadione)
AND
TS=(Stroke OR Mortality OR Death OR Bleed* OR Adverse event* OR Hemorrhage OR Haemorrhage OR thromb* OR Embol*)
AND
TS=(Randomised controlled trial OR Clinical trial OR Real-world data OR Cohort OR Observational OR Longitudinal OR Time series)

Pubmed = 3,753
(((Atrial fibrillation[Title/Abstract]) **AND** (NOAC[Title/Abstract] OR non-vitamin k oral anticoagulant[Title/Abstract] OR novel oral anticoagulant[Title/Abstract] OR DOAC[Title/Abstract] OR direct oral anticoagulant[Title/Abstract] OR Apixaban[Title/Abstract] OR Dabigatran[Title/Abstract] OR Rivaroxaban[Title/Abstract] OR Edoxaban[Title/Abstract] OR vitamin K antagonist[Title/Abstract] OR VKA[Title/Abstract] OR warfarin[Title/Abstract] OR coumadin[Title/Abstract] OR 4-hydroxycoumarin*[Title/Abstract] OR acenocoumar*[Title/Abstract] OR acenocumar*[Title/Abstract] OR coumarin*[Title/Abstract] OR cumarin*[Title/Abstract] OR ethyl biscoumacetate[Title/Abstract] OR phenprocoum*[Title/Abstract] OR phenprocum*[Title/Abstract] OR dicoumar*[Title/Abstract] OR dicumar*[Title/Abstract] OR fluindione[Title/Abstract] OR phenindione[Title/Abstract] OR clorindione[Title/Abstract] OR diphenadione[Title/Abstract])) **AND** (Stroke[Title/Abstract] OR Mortality[Title/Abstract] OR Death[Title/Abstract] OR Bleed*[Title/Abstract] OR Adverse event*[Title/Abstract] OR Hemorrhage[Title/Abstract] OR Haemorrhage[Title/Abstract] OR thromb*[Title/Abstract] OR Embol*[Title/Abstract])) **AND** (Randomised controlled trial OR Clinical trial OR Real-world data OR Cohort OR Observational OR Longitudinal OR Time series) **AND** (("2009"[Date - Publication] : "3000"[Date - Publication]))

SCOPUS = 663
(TITLE-ABS-KEY (atrial AND fibrillation) AND TITLE-ABS-KEY (noac) OR TITLE-ABS-KEY (non-vitamin AND k AND oral AND anticoagulant) OR TITLE-ABS-KEY (novel AND oral AND anticoagulant) OR TITLE-ABS-KEY (doac) OR TITLE-ABS-KEY (direct AND oral AND anticoagulant) OR TITLE-ABS-KEY (apixaban) OR TITLE-ABS-KEY (dabigatran) OR TITLE-ABS-KEY (rivaroxaban) OR TITLE-ABS-KEY (edoxaban) OR TITLE-ABS-KEY (vitamin AND k AND antagonist) OR TITLE-ABS-KEY (vka) OR TITLE-ABS-KEY (warfarin) AND TITLE-ABS-KEY (coumadin) OR TITLE-ABS-KEY (4-hydroxycoumarin*) OR TITLE-ABS-KEY (acenocoumar*) OR TITLE-ABS-KEY (acenocumar*) OR TITLE-ABS-KEY (coumarin*) OR TITLE-ABS-KEY (cumarin*) OR TITLE-ABS-KEY (ethyl AND biscoumacetate) OR TITLE-ABS-KEY (phenprocoum*) OR TITLE-ABS-KEY (phenprocum*) OR TITLE-ABS-KEY (dicoumar*) OR TITLE-ABS-KEY (dicumar*) OR TITLE-ABS-KEY (fluindione) OR

TITLE-ABS-KEY (phenindione) OR TITLE-ABS-KEY (clorindione) OR TITLE-ABS-KEY (diphenadione) AND TITLE-ABS-KEY (stroke) OR TITLE-ABS-KEY (mortality) OR TITLE-ABS-KEY (death) OR TITLE-ABS-KEY (bleed*) OR TITLE-ABS-KEY (adverse AND event*) OR TITLE-ABS-KEY (hemorrhage) OR TITLE-ABS-KEY (haemorrhage) AND TITLE-ABS-KEY (thromb*) OR TITLE-ABS-KEY (embol*)) AND PUBYEAR > 2008

Supplement S2.

Table S1. Summary of study characteristics including the study design, intervention and comparator details, outcomes, and overall summary of findings in terms of impact of apixaban vs. comparator (other DOACs or VKAs).

**First author, Year, country**<br><br>**Study design**	**Apixaban**<br>*n*=participants<br>mean (±SD) Age (years)<br>% Female/Male<br>Charlson comorbidity index<br>CHA_2DS_2-VASc score<br>HAS-BLED score	**Comparator(s)**<br>*n*=patients<br>mean (±SD) Age (years)<br>% Female/Male<br>Charlson comorbidity index<br>CHA_2DS_2-VASc score<br>HAS-BLED score	**Outcomes**	**Follow-up**	**Impact of Apixaban**<br><br>+ Favours Apixaban<br>- Favours comparator<br>= No difference / uncertain
Abraham, 2017, USA [59]<br><br>Retrospective cohort study	6,542 AF patients on Apixaban<br>Mean age 72.2 (±11.1)<br>46% Female<br><br>6,565 AF patients on Apixaban<br>Mean age 72.3 (±11.1)<br>46% Female	6,542 AF patients on Dabigatran<br>Mean age 72.1 (±10.5)<br>46% Female<br><br>6,565 AF patients on Rivaroxaban<br>Mean age 72.1 (±11.2)<br>46% Female	GI bleed	Apixaban = 89 days<br>Dabigatran = 120 days<br><br>Apixaban = 89 days<br>Rivaroxaban = 106 days	+
Adeboyeje, 2017, USA 48<br><br>Retrospective cohort study	3,689 AF patients on Apixaban<br>Mean age 69<br>41% Female	23,431 AF patients on Warfarin<br>Mean age 73<br>44% Female<br><br>8,539 AF patients on Dabigatran<br>Mean age 66<br>35% Female<br><br>8,398 AF patients on Rivaroxaban<br>Mean age 67<br>39% Female	Time to first major bleeding-related hospitalisation.<br>Major GI bleeding<br>Major Intracranial bleeding	Apixaban = 139 days<br>Warfarin = 285 days<br>Dabigatran = 212 days<br>Rivaroxaban = 169 days	+
Al-Khalili, 2016, Sweden 45	251 AF patients on Apixaban<br>Mean age 73 (±8)	233 AF patients on Dabigatran<br>Mean age 72 (±8)	Arterial thromboembolic events	Apixaban = 348 days (IQR 267-419)	=

**First author, Year, country**<br><br>**Study design**	**Apixaban**<br>*n*=participants<br>mean (±SD) Age (years)<br>% Female/Male<br>Charlson comorbidity index<br>CHA_2DS_2-VASc score<br>HAS-BLED score	**Comparator(s)**<br>*n*=patients<br>mean (±SD) Age (years)<br>% Female/Male<br>Charlson comorbidity index<br>CHA_2DS_2-VASc score<br>HAS-BLED score	**Outcomes**	**Follow-up**	**Impact of Apixaban**<br><br>+ Favours Apixaban<br>- Favours comparator<br>= No difference / uncertain
Retrospective cohort study	49% Female	49% Female<br><br>282 AF patients on Rivaroxaban<br>Mean age 73 (±8)<br>50% Female	All-cause mortality<br>Major bleed	Dabigatran = 367 days (IQR 183-493)<br>Rivaroxaban = 432 days (IQR 255-546)	
Alcusky, 2020, USA 21<br><br>Retrospective	2,881 AF patients on Apixaban<br>Median age 84 (77-89)<br>69% female<br>Median CHA_2DS_2-VASc 5 (4-6)	2,881 AF patients on Warfarin<br>Median age 84 (76-79)<br>68% female<br>Median CHA_2DS_2-VASc 5 (4-6)	Ischemic stroke/TIA<br>Ischemic stroke<br>Bleeding<br>AMI/VTE/SE<br>Mortality	Follow-up continued until a study outcome	=
Amin, 2017, USA 60<br><br>Retrospective with PSM	20,803 Apixaban-Warfarin pairs.<br>Apixaban:<br>Mean age 78.4 (±7.4)<br>52% female<br>Mean Deyo-Charlson 2.8 (±2.6)<br>Mean CHA_2DS_2-VASc 4.6 (±1.7)<br>Mean HAS-BLED 3.3 (±1.2)	20,803 apixaban-warfarin pairs.<br>Warfarin:<br>Mean age 78 (±7.5)<br>52% female<br>Mean Deyo-Charlson 2.9 (±2.6)<br>Mean CHA_2DS_2-VASc 4.7 (±1.7)<br>Mean HAS-BLED 3.3 (±1.2)	Stroke/SE<br>Major bleeding	Apixaban = 171.2 days<br>Warfarin = 196.2 days	+
Amin 2018, USA 61<br><br>Retrospective with PSM	8,328 Apixaban-warfarin pairs:<br>Apixaban:<br>Mean age 73.5 (±10.7)<br>45% Female<br>Mean Charlson 2.4 (±2.3)<br>Mean CHA_2DS_2-VASc 3.9 (±1.8)<br>Mean HAS-BLED 2.81 (±1.31)<br><br>3,557 Apixaban-Dabigatran pairs. Apixaban:<br>Mean age 70.9 (±11.4)	8,328 Apixaban-warfarin pairs:<br>Warfarin:<br>Mean age 73.4 (±10.4)<br>46% Female<br>Mean Charlson 2.36 (±2.33)<br>Mean CHA_2DS_2-VASc 3.9 (±1.8)<br>Mean HAS-BLED 2.8 (±1.3)<br><br>3,557 Apixaban-Dabigatran pairs. Dabigatran:<br>Mean age 70.7 (±11.2)	All-cause hospitalisation<br>Hospitalisations due to stroke and major bleeding	12-months	+

First author, Year, country<br><br>Study design	Apixaban<br>*n*=participants<br>mean (±SD) Age (years)<br>% Female/Male<br>Charlson comorbidity index<br>CHA_2DS_2-VASc score<br>HAS-BLED score	Comparator(s)<br>*n*=patients<br>mean (±SD) Age (years)<br>% Female/Male<br>Charlson comorbidity index<br>CHA_2DS_2-VASc score<br>HAS-BLED score	Outcomes	Follow-up	Impact of Apixaban<br><br>+ Favours Apixaban<br>- Favours comparator<br>= No difference / uncertain
	39% Female<br>Mean Charlson 2.0 (±2.2)<br>Mean CHA_2DS_2-VASc 3.5 (±1.8)<br>Mean HAS-BLED 2.56 (±1.28)<br><br>8,440 apixaban-rivaroxaban pairs. Apixaban:<br>Mean age 72.8 (±11.1)<br>44% Female<br>Mean Charlson 2.3 (±2.3)<br>Mean CHA_2DS_2-VASc 3.8 (±1.8)<br>Mean HAS-BLED 2.75 (±1.30)	30% Female<br>Mean Charlson 2.0 (±2.1)<br>Mean CHA_2DS_2-VASc 3.5 (±1.8)<br>Mean HAS-BLED 2.6 (±1.3)<br><br>8,440 apixaban-rivaroxaban pairs. Rivaroxaban:<br>Mean age 72.5 (±10.8)<br>44% Female<br>Mean Charlson 2.3 (±2.3)<br>Mean CHA_2DS_2-VASc 3.8 (±1.8)<br>Mean HAS-BLED 2.7 (±1.3)			
Amin, 2019a, USA 62<br><br>Retrospective with PSM	37,525 patients on Apixaban<br>Mean age 78.4 (±7.5)<br>52% Female<br>Mean Charlson 2.9 (±2.6)<br>Mean CHA_2DS_2-VASc 4.6 (±1.8)<br>Mean HAS-BLED 3.4 (±1.3)	37,525 patients on Warfarin<br>Mean age 78.4 (±7.4)<br>52% Female<br>Mean Charlson 2.9 (±2.7)<br>Mean CHA_2DS_2-VASc 4.7 (±1.7)<br>Mean HAS-BLED 3.4 (±1.3)	Stroke/SE<br>Major bleeding<br>Net clinical outcome<br>MACE	Mean duration 8-10 months	+
Amin, 2019b, USA 63<br><br>Retrospective with PSM	Apixaban vs Warfarin<br>10,570 patients on Apixaban<br>Mean age 80.4<br>54% Female<br>Mean CHA_2DS_2-VASC 5.4<br>Mean HAS-BLED 3.7<br>Mean Charlson 5.7<br><br>Apixaban vs Dabigatran<br>4,263 patients on Apixaban<br>Mean age 78.7	Apixaban vs Warfarin<br>10,570 patients on Warfarin<br>Mean age 80.4<br>54% Female<br>Mean CHA_2DS_2-VASC 5.4<br>Mean HAS-BLED 3.7<br>Mean Charlson 5.7<br><br>Apixaban vs Dabigatran<br>4,263 patients on Dabigatran<br>Mean age 78.8	Stroke / SE<br>Ischaemic stroke<br>Haemorrhagic stroke<br>Major bleeding<br>GI bleeding<br>Intracranial haemorrhage<br>Other bleeding<br>Major adverse cardiac event (MACE)	Mean 6-8 months across all cohorts	+

First author, Year, country<br><br>**Study design**	**Apixaban**<br>*n*=participants<br>mean (±SD) Age (years)<br>% Female/Male<br>Charlson comorbidity index<br>CHA_2DS_2-VASc score<br>HAS-BLED score	**Comparator(s)**<br>*n*=patients<br>mean (±SD) Age (years)<br>% Female/Male<br>Charlson comorbidity index<br>CHA_2DS_2-VASc score<br>HAS-BLED score	**Outcomes**	**Follow-up**	**Impact of Apixaban**<br><br>+ Favours Apixaban<br>- Favours comparator<br>= No difference / uncertain
	52% Female<br>Mean CHA_2DS_2-VASC 5.2<br>Mean HAS-BLED 3.5<br>Mean Charlson 5.2<br><br>Apixaban vs Rivaroxaban<br>10,477 patients on Apixaban<br>Mean age 80.4<br>54% Female<br>Mean CHA_2DS_2-VASC 5.4<br>Mean HAS-BLED 3.7<br>Mean Charlson 5.7	51% Female<br>Mean CHA_2DS_2-VASC 5.2<br>Mean HAS-BLED 3.5<br>Mean Charlson 5.3<br><br>Apixaban vs Rivaroxaban<br>10,477 patients on Rivaroxaban<br>Mean age 80.3<br>54% Female<br>Mean CHA_2DS_2-VASC 5.4<br>Mean HAS-BLED 3.7<br>Mean Charlson 5.7	Myocardial infarction<br>All-cause mortality		
Andersson, 2018, Denmark 64<br><br>Retrospective with PSM	Apixaban vs Dabigatran<br>3,235 patients on Apixaban<br>63% Male<br>Mean age 67.6 (±8.2)<br><br>Apixaban vs Rivaroxaban<br>3,676 patients on Apixaban<br>56% Male<br>Mean age 71.9 (±9.1)	Apixaban vs Dabigatran<br>3,235 patients on Dabigatran<br>64% Male<br>Mean age 67.5 (±7.3)<br><br>Apixaban vs Rivaroxaban<br>3,676 patients on Rivaroxban<br>56% Male<br>Mean age 72.0 (±9.8)	Stroke / SE<br>Major bleeding	Apixaban vs Dabigatran:<br>Apixaban mean 210 days<br>Dabigatran mean 241 days<br><br>Apixaban vs Rivaroxaban<br>Apixaban mean 212 days<br>Rivaroxaban mean 201 days	=
Bang, 2020, Korea 24<br><br>Retrospective with Inverse Probability of Treatment Weighting	10,548 patients on Apixaban<br>Mean age 71.6<br>44% Female<br>Mean CHADSVACS 4.5<br>Mean HAS-BLED 3.5<br>Mean Charlson 4.3	8,648 patients on Warfarin<br>Mean age 71.6<br>44% Female<br>Mean CHA_2DS_2-VASC 4.5<br>Mean HAS-BLED 3.5<br>Mean Charlson 4.3	Stroke / SE<br>Major bleeding<br>Intracranial haemorrhage<br>GI bleeding	Median 149 days Apixaban<br>Median 105 days Warfarin	+
Bradley, 2020, USA 52	55,038 patients on Apixaban<br>Mean age 71.3 (±9.7)	55,038 patients on Warfarin<br>Mean age 71.3 ±10.6	GI bleeding<br>Intracranial	NR	+

First author, Year, country<br><br>**Study design**	**Apixaban**<br>*n*=participants<br>mean (±SD) Age (years)<br>% Female/Male<br>Charlson comorbidity index<br>CHA_2DS_2-VASc score<br>HAS-BLED score	**Comparator(s)**<br>*n*=patients<br>mean (±SD) Age (years)<br>% Female/Male<br>Charlson comorbidity index<br>CHA_2DS_2-VASc score<br>HAS-BLED score	**Outcomes**	**Follow-up**	**Impact of Apixaban**<br><br>+ Favours Apixaban<br>- Favours comparator<br>= No difference / uncertain
Retrospective with PSM	61% Male<br>Mean CHA_2DS_2-VASC 3.2 (±1.5)<br>Mean HAS-BLED 2.2 ±1.0	61% Male<br>Mean CHA_2DS_2-VASC 3.2 ±1.5<br>Mean HAS-BLED 2.2 ±1.1	haemorrhage<br>Ischaemic stroke	Person years at risk approximately 20,000 for Apixaban and 25,000 for Warfarin	
Chan, 2018, Taiwan 25<br><br>Retrospective with PSM (unmatched baseline data given as matched cohort n= not stated)	5,843 patients on Apixaban<br>Mean age 76 (±10)<br>45% Female<br>Mean CHA_2DS_2-VASC 3.9 (±1.6)<br>Mean HAS-BLED 3.0 (±1.1)	19,375 patients on Warfarin<br>Mean age 76 (±10)<br>46% Female<br>Mean CHA_2DS_2-VASC 3.9 (±0.9)<br>Mean HAS-BLED 3.0 (±0.6)	Ischaemic stroke / SE<br>Acute MI<br>All-cause mortality<br>Intracranial haemorrhage<br>GI bleeding<br>Major bleeding	Apixaban = 0.76 years<br><br>Warfarin = 1.47 years	+
Coleman, 2016, USA 65<br><br>Retrospective with PSM	4,083 patients on Apixaban<br>Mean age 71.0 (±11.3)<br>53% Male<br>Mean CHA_2DS_2-VASC 3.5 (±1.4)<br>Mean HAS-BLED 1.7 (±0.7)<br>Mean Charlson 1.1 (±1.1)	4,083 patients on Warfarin<br>Mean age 71.2 (±11.3)<br>54% Male<br>Mean CHA_2DS_2-VASC 3.5 (±1.4)<br>Mean HAS-BLED 1.7 (±0.7)<br>Mean Charlson 1.0 (±1.1)	Intracranial haemorrhage<br>Ischaemic stroke<br>Combined ICH / IS	Mean follow-up not given<br><br>Apixaban = 2125 person-years<br><br>Warfarin = 1951 person-years	+
Coleman, 2017, USA 66<br><br>Retrospective with PSM	1,257 AF patients on Apixaban with prior stroke/TIA<br>Median age 74 (IQR 63, 82)<br>Male 54%<br>Median CHA_2DS_2-VASc 5 (IQR 4,6)<br>Median HAS-BLED 4 (IQR 4, 5)<br>Median Charlson 3 (IQR 3,4)	1,257 AF patients on Warfarin with prior stroke/TIA<br>Median age 74 (IQR 63, 82)<br>Male 56%<br>Median CHA_2DS_2-VASc 5 (IQR 4,6)<br>Median HAS-BLED 4 (IQR 4, 5)<br>Median Charlson 3 (IQR 3,4)	Ischaemic stroke<br>ICH<br>Major bleed	6-months	=
Coleman, 2018, Germany 67	835 AF patients on Apixaban<br>Mean age 75.3 (±10.6)<br>50% Male	835 AF patients on VKA<br>Mean age 74.8 (±9.2)<br>53% Male	Ischaemic stroke<br>TIA<br>MI	12-months	=

**First author, Year, country**<br><br>**Study design**	**Apixaban**<br>*n*=participants<br>mean (±SD) Age (years)<br>% Female/Male<br>Charlson comorbidity index<br>CHA_2DS_2-VASc score<br>HAS-BLED score	**Comparator(s)**<br>*n*=patients<br>mean (±SD) Age (years)<br>% Female/Male<br>Charlson comorbidity index<br>CHA_2DS_2-VASc score<br>HAS-BLED score	**Outcomes**	**Follow-up**	**Impact of Apixaban**<br><br>+ Favours Apixaban<br>- Favours comparator<br>= No difference / uncertain
Retrospective with PSM	Mean CHA_2DS_2-VASc 3.5 (±1.5)<br>>1 relevant co-morbidities 74%	Mean CHA_2DS_2-VASc 3.6 (±1.5)<br>≥1 relevant co-morbidities 78%			
Deitelzweig, 2016, USA 68<br><br>Retrospective with multivariate regression	4,138 AF patients on Apixaban<br>Mean age 73.6 (±11.6)<br>51% Female<br>Mean Charlson 2.1 (±1.2)<br>Mean CHA_2DS_2-VASc 3.7 (±1.6)<br>HAS-BLED mean 2.4 (±1.0)	37,754 AF patients on rivaroxaban<br>Mean age 72.3 (±11.8)<br>49% Female<br>Mean Charlson 2.1 (±1.1)<br>Mean CHA_2DS_2-VASc 3.7 (±1.6)<br>Mean HAS-BLED 2.4 (±1.0)<br><br>32,838 AF patients on dabigatran<br>Mean age 71.9 (±11.8)<br>46% Female<br>Mean Charlson 2.1 (±1.9)<br>Mean CHA_2DS_2-VASc 3.7 (±1.6)<br>Mean HAS-BLED 2.3 (±1.0)	Bleeding-related hospital readmission<br>All-cause hospital readmission	1-month	+
Deitelzweig, 2017, USA 69<br><br>Retrospective with PSM	6,810 AF patients on Apixaban<br>Mean age 77.1 (±8.0)<br>48% Female<br>Mean Charlson 2.8 (±2.3)<br>Mean CHA_2DS_2-VASc 4.4 (±1.6)<br>Mean HAS-BLED 2.9 (±1.1)	Apixaban vs Rivaroxaban<br>After PSM, 6,810 AF patients were in each cohort. Mean ages (77.1 vs. 77.0 years), Charlson (2.8 vs. 2.7), CHA_2DS_2-VASc (4.4 vs. 4.4), HAS-BLED (2.9 vs. 2.9).<br><br>Apixaban vs Dabigatran<br>After PSM, 2,327 AF patients were in each cohort. Mean ages (77.3 vs. 76.9 years), Charlson	Stroke/SE<br>Ischaemic stroke<br>Haemorrhagic stroke<br>SE<br>Major bleeding<br>GI bleeding	6-months	+

**First author, Year, country**<br><br>**Study design**	**Apixaban**<br>*n*=participants<br>mean (±SD) Age (years)<br>% Female/Male<br>Charlson comorbidity index<br>CHA_2DS_2-VASc score<br>HAS-BLED score	**Comparator(s)**<br>*n*=patients<br>mean (±SD) Age (years)<br>% Female/Male<br>Charlson comorbidity index<br>CHA_2DS_2-VASc score<br>HAS-BLED score	**Outcomes**	**Follow-up**	**Impact of Apixaban**<br><br>+ Favours Apixaban<br>- Favours comparator<br>= No difference / uncertain
		(2.6 vs. 2.6), CHA_2DS_2-VASc (4.6 vs. 4.3), HAS-BLED (2.9 vs. 2.9).<br><br>Apixaban vs Warfarin<br>After PSM, 7,107 AF patients were in each cohort. Mean ages (78.2 vs. 78.1 years), Charlson (3.0 vs. 3.0), CHA_2DS_2-VASc (4.6 vs. 4.6), HAS-BLED (3.0 vs. 3.1).			
Durand, 2020, Canada, USA, UK 70<br><br>Retrospective with PSM	Apixaban Vs Dabigatran<br>49,058 pairs<br>Mean age 75.3<br>54% Male<br><br>Apixaban Vs Rivaroxaban<br>54,276 pairs<br>Mean age 75.5<br>54% Male	NA	Ischaemic stroke or SE<br>Major bleeding<br>MI<br>Intracranial bleeding<br>GI bleeding<br>All-cause mortality	12-months	+
Fralick, 2020, USA 42<br><br>Retrospective with PSM	39,351 AF patients on Apixaban<br>Mean age 69.4 (±10.5)<br>40% Female	39,351 AF patients on Rivaroxaban<br>Mean age 69.3 (±10.6)<br>40% Female	Stroke<br>SE<br>GI bleeding<br>ICH	Apixaban = Mean follow-up 288 days<br>Rivaroxaban = Mean follow-up 291 days	+
Graham, 2019, USA 26<br><br>Retrospective with Inverse Probability of Treatment Weighting	72,921 AF patients on Apixaban<br>Mean age 75.1<br>47% Female<br>80% had CHA_2DS_2-VASc $\geq$3<br>45% had HAS-BLED $\geq$3	183,003 AF patients on Warfarin<br>Mean age 75.2<br>47% Female<br>80% had CHA_2DS_2-VASc $\geq$3<br>45% had HAS-BLED $\geq$3	Thromboembolic stroke<br>ICH<br>All-cause mortality	A total of 448,944 anticoagulant initiators contributed 159,927 person-years of on-treatment follow-up (mean duration 130 days).	+

First author, Year, country<br><br>Study design	Apixaban<br>*n*=participants<br>mean (±SD) Age (years)<br>% Female/Male<br>Charlson comorbidity index<br>CHA_2DS_2-VASc score<br>HAS-BLED score	Comparator(s)<br>*n*=patients<br>mean (±SD) Age (years)<br>% Female/Male<br>Charlson comorbidity index<br>CHA_2DS_2-VASc score<br>HAS-BLED score	Outcomes	Follow-up	Impact of Apixaban<br><br>+ Favours Apixaban<br>- Favours comparator<br>= No difference / uncertain
		86,293 AF patients on Dabigatran<br>Mean age 75.1<br>47% Female<br>80% had CHA_2DS_2-VASc ≥3<br>45% had HAS-BLED ≥3<br><br>106,369 AF patients on Dabigatran<br>Mean age 75.1<br>47% Female<br>81% had CHA_2DS_2-VASc ≥3<br>45% had HAS-BLED ≥3			
Granger, 2011, International (39 countries)[13]<br><br>Double blind RCT	9,120 AF patients on Apixaban<br>Median age 70 (IQR 63-76)<br>36% Female	9,081 AF patients on Warfarin<br>Median age 70 (IQR 63-76)<br>35% Female	Stroke<br>Haemorrhagic stroke<br>SE<br>All-cause mortality	1.8 years	+
Gupta, 2019, USA [71]<br><br>Retrospective with PSM	7,607 AF patients on Apixaban<br>Mean age 76.5 (±9.5)<br>58% Male<br>Charlson 2.5 (±2.4)<br>CHA_2DS_2-VASc 4.2 (±1.8)<br>HAS-BLED 3.0 (±1.3)	7,607 AF patients on Warfarin<br>Mean age 76.6 (±9.8)<br>58% Male<br>Charlson 2.5 (±2.4)<br>CHA_2DS_2-VASc 4.1 (±1.8)<br>HAS-BLED 3.0 (±1.3)	Ischaemic stroke<br>Haemorrhagic stroke<br>SE<br>Major bleeding<br>GI Bleeding<br>ICH	Apixaban = 161 days<br>Warfarin = 153 days<br><br>Results given as incidence rates /100 PY	+
Halvorsen, 2017, Norway [72]	6,506 AF patients on Apixaban<br>Mean age 74.5<br>55% Male<br>≥1 comorbidity 60%	11,427 AF patients on Warfarin<br>Mean age 74.6<br>59% Male<br>≥1 comorbidity 66%	Major bleed<br>Clinically relevant non-major bleed	Mean follow-up of 173 days	+

**First author, Year, country**<br><br>**Study design**	**Apixaban**<br>*n*=participants<br>mean (±SD) Age (years)<br>% Female/Male<br>Charlson comorbidity index<br>CHA_2DS_2-VASc score<br>HAS-BLED score	**Comparator(s)**<br>*n*=patients<br>mean (±SD) Age (years)<br>% Female/Male<br>Charlson comorbidity index<br>CHA_2DS_2-VASc score<br>HAS-BLED score	**Outcomes**	**Follow-up**	**Impact of Apixaban**<br><br>+ Favours Apixaban<br>- Favours comparator<br>= No difference / uncertain
Prospective cohort study	CHA_2DS_2-VASc 2.9<br>HAS-BLED $\geq$3 47%	CHA_2DS_2-VASc 3.1<br>HAS-BLED >3 43%	GI bleed<br>ICH		
Hernandez, 2017, USA 73<br><br>Retrospective	2,358 AF patients on Apixaban<br>Mean age 77.4 ±8.6<br>43% Male<br>CHA_2DS_2-VASc 4.7 (±1.7)<br>HAS-BLED 3.7 (±0.9)	12,353 AF patients on Warfarin<br>Mean age 78.0 (±11.0)<br>41% Male<br>CHA_2DS_2-VASc 4.8 (±1.9)<br>HAS-BLED 3.8 (±1.0)<br><br>1,415 AF patients on Dabigatran<br>Mean age 74.9 (±8.7)<br>47% Male<br>CHA_2DS_2-VASc 4.3 (±1.7)<br>HAS-BLED 3.5 (±0.9)<br><br>5,139 AF patients on Rivaroxaban<br>Mean age 76.4 (±8.6)<br>44% Male<br>CHA_2DS_2-VASc 4.6 (±1.8)<br>HAS-BLED 3.7 (±1.0)	Ischaemic stroke<br>All-cause mortality<br>Any bleeding event<br>Intracranial bleeding<br>GI bleeding	Apixaban = 185 days<br>Dabigatran = 294 days<br>Rivaroxaban = 255 days<br>Warfarin = 274 days	+
Hohnloser, 2017, Germany 27<br><br>Retrospective with adjusted HRs	3,633 AF patients on Apixaban<br>Mean age 75.5 (±10.8)<br>49% Male<br>Carlson 3.4 (±2.7)<br>CHA_2DS_2-VASc 4.1 (±1.8)<br>HAS-BLED 2.9 (±1.2)	16,179 AF patients on Phenprocoumon<br>Mean age 76.1 (±9.1)<br>50% Male<br>Carlson 3.4 (±2.6)<br>CHA_2DS_2-VASc 4.1 (±1.6)<br>HAS-BLED 2.7 (±1.1)	Major bleeding<br>GI bleeding<br>Any bleeding	Mean Apixaban follow-up 218 days<br>Mean Phenprocoumon follow-up 280 days	+
Hohnloser, 2018, Germany 74	10,117 AF patients on Apixaban<br>Mean age 74.5 (±11.4) years	23,823 AF patients on Phenprocoumon	Stroke	Phenprocoumon = 362 days	+

First author, Year, country<br><br>Study design	Apixaban<br>*n*=participants<br>mean (±SD) Age (years)<br>% Female/Male<br>Charlson comorbidity index<br>CHA_2DS_2-VASc score<br>HAS-BLED score	Comparator(s)<br>*n*=patients<br>mean (±SD) Age (years)<br>% Female/Male<br>Charlson comorbidity index<br>CHA_2DS_2-VASc score<br>HAS-BLED score	Outcomes	Follow-up	Impact of Apixaban<br><br>+ Favours Apixaban<br>- Favours comparator<br>= No difference / uncertain
 Retrospective with adjusted HRs	51% Male<br>Charlson 3.4 (±2.7)<br>CHA_2DS_2-VASc 4.0 (±1.8)<br>HAS-BLED 2.8 (±1.2)	Mean age 75.2 (±9.5) years<br>53% Male<br>Charlson 3.4 (±2.6)<br>CHA_2DS_2-VASc 4.0 (±1.6)<br>HAS-BLED 2.8 (±1.1)<br><br>5,122 AF patients on Dabigatran<br>Mean age 71.7 (±11.6) years<br>55% Male<br>Charlson 2.9 (±2.5)<br>CHA_2DS_2-VASc 3.7 (±1.8)<br>HAS-BLED 2.6 (±1.2)<br><br>22,143 AF patients on Rivaroxaban<br>Mean age 72.1 (11.8) years<br>55% Male<br>Charlson 2.9 (±2.5)<br>CHA_2DS_2-VASc 3.5 (±1.8)<br>HAS-BLED 2.5 (±1.2)	Ischaemic stroke<br>Haemorrhagic stroke<br>All-cause mortality<br>Major bleeding<br>Intracranial bleeding<br>GI bleeding	Apixaban = 306<br>Dabigatran = 339 days<br>Rivaroxaban = 340 days	
Huybrechts, 2020, USA 53<br><br>Retrospective Cohort with PSM	19,588 AF patients on Apixaban<br><br>Characteristics not given as this was a secondary outcome	19,588 patients on Warfarin<br><br><br>Characteristics not given as this was a secondary outcome	Stroke<br>Major haemorrhage	5 years	+
Jansson, 2020, Sweden 39	11,493 AF patients on Apixaban<br>Mean age 73.5 (±10.3)<br>57% Male	6,453 AF patients on Dabigatran<br>Mean age 72.3 (±9.8)<br>58% Male	Ischaemic stroke<br>Combined all cause stroke + SE<br>Myocardial infarction	Apixaban - Median 205 days (IQR 83-381)<br><br>Dabigatran - Median 352	=

First author, Year, country<br><br>**Study design**	**Apixaban**<br>*n*=participants<br>mean (±SD) Age (years)<br>% Female/Male<br>Charlson comorbidity index<br>CHA_2DS_2-VASc score<br>HAS-BLED score	**Comparator(s)**<br>*n*=patients<br>mean (±SD) Age (years)<br>% Female/Male<br>Charlson comorbidity index<br>CHA_2DS_2-VASc score<br>HAS-BLED score	**Outcomes**	**Follow-up**	**Impact of Apixaban**<br><br>+ Favours Apixaban<br>- Favours comparator<br>= No difference / uncertain
Retrospective Cohort with full optimal matching		7,897 AF patients on Rivaroxaban<br>Mean age 73.5 ± 10.3<br>57% Male	All-cause mortality<br>Haemorrhagic stroke<br>Major bleeding	days (IQR 111-750)<br><br>Rivaroxaban - Median 267 (IQR 102-532)	
Kjerpeseth, 2019, Norway 28<br><br>Retrospective Cohort	10,550 AF patients on Apixaban<br>Mean age 74.2 (±11.0)<br>46% Female<br>Mean CHA_2DS_2-VASC 3.5 (±1.7)<br>Mean HAS-BLED 2.5 (±1.1)	6,435 AF patients on Warfarin<br>Mean age 73.6 (±11.9)<br>41% Female<br>Mean CHA_2DS_2-VASC 3.5 (± 1.8)<br>Mean HAS-BLED 2.6 (±1.2)	Ischaemic stroke, TIA or SE<br>Ischaemic stroke or SE<br>Ischaemic stroke<br>TIA<br>Major (or clinically relevant non-major) bleeding<br>Intracranial bleeding<br>GI bleeding	365 days or censored at change of treatment	+
Kohsaka, 2017, Japan 29<br><br>Retrospective cohort with PSM	5,977 AF patients on Apixaban<br>Mean age 77.4 (±10)<br>41% Female<br>Mean CHA_2DS_2-VASC 3.5 (±1.6)	5,977 AF patients on Warfarin<br>Mean age 77.7 (±10)<br>41% Female<br>Mean CHA_2DS_2-VASC 3.5 (±1.5)	Major bleeding<br><br>Any bleeding	Results given as rate per 100 patient-years only given.<br><br>Average follow-up per group NR.	+
Kohsaka, 2018, Japan 75<br><br>Retrospective cohort with PSM	11,972 AF patients on Apixaban<br>Mean age 77.6 (±10.0)<br>42% Female<br>Mean CHA_2DS_2-VASC 3.4 (±1.6)	11,972 AF patients on Warfarin<br>Mean age 77.7 (±10.0)<br>42% Female<br>Mean CHA_2DS_2-VASC 3.4 (±1.5)	Major bleeding<br>Any bleeding<br>Stroke or SE	Apixaban = 123 days<br>Warfarin = 110 days	+
Lamberts, 2017, Denmark 49	7,963 AF patients on Apixaban<br>Mean age 75.4 (±11.1)<br>51% Male	6,715 AF patients on Rivaroxaban<br>Mean age 74.4 (± 11.0)<br>52% Male	Major bleeding (total)<br>Major bleeding (30 day)	Apixaban = 268.1 days<br>Rivaroxaban = 348.5 days<br>Dabigatran = 511.4 days<br>Warfarin = 398.0 days	+

First author, Year, country<br><br>Study design	Apixaban<br>*n*=participants<br>mean (±SD) Age (years)<br>% Female/Male<br>Charlson comorbidity index<br>CHA_2DS_2-VASc score<br>HAS-BLED score	Comparator(s)<br>*n*=patients<br>mean (±SD) Age (years)<br>% Female/Male<br>Charlson comorbidity index<br>CHA_2DS_2-VASc score<br>HAS-BLED score	Outcomes	Follow-up	Impact of Apixaban<br><br>+ Favours Apixaban<br>- Favours comparator<br>= No difference / uncertain
Retrospective cohort with adjusted HRs	Mean CHA_2DS_2-VASC 3.2 (±1.6)<br>Mean HAS-BLED 2.3 (±1.2)	Mean CHA_2DS_2-VASC 3.0 (±1.6)<br>Mean HAS-BLED 2.2 (±1.2)<br><br>15,413 AF patients on Dabigatran<br>Mean age 71.5 (±11.0)<br>57% Male<br>Mean CHA_2DS_2-VASC 2.7 (±1.6)<br>Mean HAS-BLED 2.1 (±1.2)<br><br>24,230 AF patients on Warfarin<br>Mean age 72.1 (±11.3)<br>58% Male<br>Mean CHA_2DS_2-VASC 2.9 (±1.7)<br>Mean HAS-BLED 2.2 (±1.2)		Overall mean = 403 days	
Larsen, 2016, Denmark 30<br><br>Retrospective cohort with Inverse Probability of Treatment Weighting	6,349 AF patients on Apixaban<br>Median age 71.3 (IQR 65.8 - 77.2)<br>40% Female<br>Mean CHA_2DS_2-VASC 2.8 (±1.6)<br>Mean HAS-BLED 2.3 (±19.2)	35,436 AF patients on Warfarin<br>Median age 70.9 (IQR 64.3 - 77.7)<br>40% Female<br>Mean CHA_2DS_2-VASC 2.7 (±1.6)<br>Mean HAS-BLED 2.2 (±1.2)	Ischaemic stroke or SE<br>Ischaemic stroke<br>All-cause mortality<br>Any bleeding<br>Major bleeding<br>Intracranial bleeding	1 year	+
Lee, 2019, Korea 23<br><br>Retrospective cohort with Inverse Probability of Treatment Weighting	Apixaban vs Warfarin:<br>22,177 AF patients on Apixaban<br>Mean age 70.9 (±11.0)<br>56% Male<br>Mean CHA_2DS_2-VASC 3.6 (±1.4)<br>Mean HAS-BLED 2.7 (±1.1)<br>Mean Charlson 3.8 (±2.3)	Apixaban vs Warfarin:<br>25,420 AF patients on Warfarin<br>Mean age 71.2 (±11.1)<br>55% Male<br>Mean CHA_2DS_2-VASC 3.6 (±1.6)<br>Mean HAS-BLED 2.7 (±1.1)<br>Mean Charlson 3.82 ± 2.39	Ischaemic stroke<br>Intracranial haemorrhage<br>GI bleeding<br>Major bleeding	Apixaban = 0.80 years<br>Rivaroxaban = 0.87 years<br>Dabigatran = 0.87 years<br>Edoxaban = 0.57 years<br>Warfarin = 0.82 years	+

**First author, Year, country**<br><br>**Study design**	**Apixaban**<br>*n*=participants<br>mean (±SD) Age (years)<br>% Female/Male<br>Charlson comorbidity index<br>CHA_2DS_2-VASc score<br>HAS-BLED score	**Comparator(s)**<br>*n*=patients<br>mean (±SD) Age (years)<br>% Female/Male<br>Charlson comorbidity index<br>CHA_2DS_2-VASc score<br>HAS-BLED score	**Outcomes**	**Follow-up**	**Impact of Apixaban**<br><br>+ Favours Apixaban<br>- Favours comparator<br>= No difference / uncertain
	Apixaban vs Rivaroxaban:<br>22,177 AF patients on Apixaban<br>Mean age 72.3 (±10.1)<br>53% Male<br>Mean CHA_2DS_2-VASC 3.7 (±1.4)<br>Mean HAS-BLED 2.8 (±1.0)<br>Mean Charlson 3.8 (±2.3)<br><br>Apixaban vs Dabigatran:<br>22,177 AF patients on Apixaban<br>Mean age 71.8 (±10.3)<br>54% Male<br>Mean CHA_2DS_2-VASC 3.7 (±1.4)<br>Mean HAS-BLED 2.7 (±1.03)<br>Mean Charlson 3.8 (±2.3)<br><br>Apixaban vs Edoxaban:<br>22,177 AF patients on Apixaban<br>Mean age 72.3 (±10.3)<br>53% Male<br>Mean CHA_2DS_2-VASC 3.7 (±1.4)<br>Mean HAS-BLED 2.7 (±1.0)<br>Mean Charlson 3.8 (±2.3)	Apixaban vs Rivaroxaban:<br>35,965 AF patients on Rivaroxaban<br>Mean age 72.3 (±10.0)<br>53% Male<br>Mean CHA_2DS_2-VASC 3.7 (± 1.4)<br>Mean HAS-BLED 2.8 (±1.0)<br>Mean Charlson 3.8 (±2.3)<br><br>Apixaban vs Dabigatran:<br>17,745 AF patients on Dabigatran<br>Mean age 71.8 (±9.9)<br>54% Male<br>Mean CHA_2DS_2-VASC 3.7 (±1.38)<br>Mean HAS-BLED 2.71 ± 1.02<br>Mean Charlson 3.8 (±2.3)<br><br>Apixaban vs Edoxaban:<br>15,496 AF patients on Edoxaban<br>Mean age 72.3 (±9.8)<br>53% Male<br>Mean CHA_2DS_2-VASC 3.7 (±1.40)<br>Mean HAS-BLED 2.7 (±1.0)<br>Mean Charlson 3.8 (±2.4)			

First author, Year, country<br><br>Study design	Apixaban<br>*n*=participants<br>mean (±SD) Age (years)<br>% Female/Male<br>Charlson comorbidity index<br>CHA_2DS_2-VASc score<br>HAS-BLED score	Comparator(s)<br>*n*=patients<br>mean (±SD) Age (years)<br>% Female/Male<br>Charlson comorbidity index<br>CHA_2DS_2-VASc score<br>HAS-BLED score	Outcomes	Follow-up	Impact of Apixaban<br><br>+ Favours Apixaban<br>- Favours comparator<br>= No difference / uncertain
Li, 2017, USA 31<br><br>Retrospective cohort with PSM	38,470 AF patients on Apixaban<br>Mean age 70.9 (±12.0)<br>40% Female<br>Mean CHA_2DS_2-VASC 3.2 (±1.8)<br>Mean HAS-BLED 2.6 (±1.4)<br>Mean Charlson 2.5 (±2.4)	38,470 AF patients on Warfarin<br>Mean age 70.9 (±11.9)<br>40% Female<br>Mean CHA_2DS_2-VASC 3.2 (±1.7)<br>Mean HAS-BLED 2.6 (±1.3)<br>Mean Charlson 2.5 (±2.5)	Stroke / SE<br>Ischaemic stroke<br>Haemorrhagic stroke<br>SE<br>Major bleeding<br>Intracranial haemorrhage<br>GI bleeding<br>Other bleeding	Apixaban = 179 days<br>Warfarin = 200 days	+
Lin, 2017, USA 19<br><br><br>Retrospective cohort with PSM	Apixaban vs Rivaroxaban<br>4,062 AF patients on Apixaban<br>Mean age 62.0 (±8.5)<br>29% Female<br>Mean CHA_2DS_2-VASC 2.1 (±1.4)<br>Mean HAS-BLED 2.2 (±0.9)<br>Mean Charlson 1.2 (±1.6)<br><br>Apixaban vs Dabigatran<br>2,684 AF patients on Apixaban<br>Mean age 63.0 (±9.2)<br>28% Female<br>Mean CHA_2DS_2-VASC 2.1 (±1.5)<br>Mean HAS-BLED 2.1 (±0.9)<br>Mean Charlson 1.2 (±1.6)<br><br>Apixaban vs Warfarin<br>4,847 AF patients on Apixaban<br>Mean age 63.9 (±9.5)<br>32% Female	Apixaban vs Rivaroxaban<br>4,062 AF patients on Rivaroxaban<br>Mean age 62.0 (±8.4)<br>30% Female<br>Mean CHA_2DS_2-VASC 2.1 (±1.4)<br>Mean HAS-BLED 2.2 (±0.9)<br>Mean Charlson 1.2 (±1.6)<br><br>Apixaban vs Dabigatran<br>2,684 AF patients on Dabigatran<br>Mean age 63.0 (±9.3)<br>28% Female<br>Mean CHA_2DS_2-VASC 2.1 (±1.5)<br>Mean HAS-BLED 2.1 (±0.9)<br>Mean Charlson 1.2 (±1.6)<br><br>Apixaban vs Warfarin<br>4,847 AF patients on Warfarin<br>Mean age 64.0 (±9.4)	1) Hospitalisations<br><br>2) Major bleeding	Matched cohorts (mean months ± SD)<br><br>Apixaban vs Rivaroxaban<br>Apixaban 4.5 ± 4.3<br>Rivaroxaban 4.5 ± 4.5<br><br>Apixaban vs Dabigatran<br>Apixaban 5.2 ± 5.1<br>Dabigatran 5.0 ± 5.2<br><br>Apixaban vs Warfarin<br>Apixaban 4.9 ± 4.9<br>Warfarin 4.8 ± 4.8	+

First author, Year, country<br><br>**Study design**	**Apixaban**<br>*n*=participants<br>mean (±SD) Age (years)<br>% Female/Male<br>Charlson comorbidity index<br>CHA_2DS_2-VASc score<br>HAS-BLED score	**Comparator(s)**<br>*n*=patients<br>mean (±SD) Age (years)<br>% Female/Male<br>Charlson comorbidity index<br>CHA_2DS_2-VASc score<br>HAS-BLED score	**Outcomes**	**Follow-up**	**Impact of Apixaban**<br><br>+ Favours Apixaban<br>- Favours comparator<br>= No difference / uncertain
	Mean CHA_2DS_2-VASC 2.3 (±1.6)<br>Mean HAS-BLED 2.2 (±1.0)<br>Mean Charlson 1.4 (±1.8)	31% Female<br>Mean CHA_2DS_2-VASC 2.3 (±1.7)<br>Mean HAS-BLED 2.2 (±1.1)<br>Mean Charlson 1.4 (±1.8)			
Lip, 2016a, USA 32<br><br>Retrospective Cohort with PSM	vs Warfarin cohort:<br>6,964 AF patients on Apixaban<br>Mean age 69.1 (±12.3)<br>39% Female<br>Mean CHA_2DS_2-VASC 2.9 (±1.7)<br>Mean HAS-BLED 2.2 (±1.3)<br>Mean Deyo-Charlson 1.9 (±2.0)<br><br>vs Dabigatran cohort:<br>4,407 AF patients on Apixaban<br>Mean age 67 (±12.3)<br>36% Female<br>Mean CHA_2DS_2-VASC 2.5 (±1.6)<br>Mean HAS-BLED 2.0 (±1.2)<br>Mean Deyo-Charlson 1.6 (±1.9)<br><br>vs Rivaroxaban cohort:<br>7,399 AF patients on Apixaban<br>Mean age 68.4 (±12.4)<br>39% Female<br>Mean CHA_2DS_2-VASC 2.8 (±1.6)<br>Mean HAS-BLED 2.2 (±1.2)<br>Mean Deyo-Charlson 1.8 (±2.0)	vs Warfarin cohort:<br>6,964 AF patients on Warfarin<br>Mean age 69.0 (±12.3)<br>38% Female<br>Mean CHA_2DS_2-VASC 2.8 (±1.6)<br>Mean HAS-BLED 2.2 (±1.2)<br>Mean Deyo-Charlson 1.8 (±2.0)<br><br>vs Dabigatran cohort:<br>4,407 AF patients on Dabigatran<br>Mean age 66.9 (±12.2)<br>36% Female<br>Mean CHA_2DS_2-VASC 2.6 (±1.7)<br>Mean HAS-BLED 2.0 (±1.2)<br>Mean Deyo-Charlson 1.6 (±1.9)<br><br>vs Rivaroxaban cohort:<br>7,399 AF patients on Rivaroxaban<br>Mean age 68.3 (±12.2)<br>39% Female<br>Mean CHA_2DS_2-VASC 2.8 (±1.7)<br>Mean HAS-BLED 2.1 (±1.2)<br>Mean Deyo-Charlson 1.7 (±2.0)	Major bleeding requiring hospitalisation	Apixaban vs Warfarin cohort:<br>Apixaban 148.1 days<br>Warfarin 161.6 days<br><br>Apixaban vs Dabigatran cohort:<br>Apixaban 145.6 days<br>Dabigatran 179.0 days<br><br>Apixaban vs Rivaroxaban cohort:<br>Apixaban 147.6 days<br>Rivaroxaban 182.1 days	+

**First author, Year, country**<br><br>**Study design**	**Apixaban**<br>*n*=participants<br>mean (±SD) Age (years)<br>% Female/Male<br>Charlson comorbidity index<br>CHA_2DS_2-VASc score<br>HAS-BLED score	**Comparator(s)**<br>*n*=patients<br>mean (±SD) Age (years)<br>% Female/Male<br>Charlson comorbidity index<br>CHA_2DS_2-VASc score<br>HAS-BLED score	**Outcomes**	**Follow-up**	**Impact of Apixaban**<br><br>+ Favours Apixaban<br>- Favours comparator<br>= No difference / uncertain
Lip, 2016b, USA 33<br><br>Retrospective cohort with adjusted HRs	2,402 AF patients on Apixaban<br>Mean age 69.3 (±12.3)<br>37% Female<br>Mean CHA_2DS_2-VASC 2.8 (±1.6)<br>Mean Charlson 1.9 (±2.0)	4,173 AF patients on Dabigatran<br>Mean age 66.8 (±12.8)<br>34% Female<br>Mean CHA_2DS_2-VASC 2.6 (±1.7)<br>Mean Charlson 1.7 (±2.0)<br><br>10,050 AF patients on Rivaroxaban<br>Mean age 67.3 (±12.3)<br>37% Female<br>Mean CHA_2DS_2-VASC 2.6 (±1.7)<br>Mean Charlson 1.8 (±2.0)<br><br>12,713 AF patients on Warfarin<br>Mean age 72.5 (±11.9)<br>39% Female<br>Mean CHA_2DS_2-VASC 3.2 (±1.7)<br>Mean Charlson 2.4 (±2.3)	Major bleeding	Apixaban = 90.4 days<br><br>Dabigatran = 126.7 = days<br><br>Rivaroxaban = 117.7 days<br><br>Warfarin = 127.6 days	+
Lip, 2017, Denmark 76<br><br>Retrospective with Inverse Probability of Treatment Weighting	Unweighted cohorts<br>1,470 AF patients on Apixaban<br>Median age 67.4 (IQR 62.5 - 70.9)<br>40% Female<br>Mean HAS-BLED 1.5 (±0.6)	Unweighted cohorts<br>7,674 AF patients on Warfarin<br>Median age 66.2 (IQR 60.5 - 70.4)<br>36% Female<br>Mean HAS-BLED 1.5 (±0.7)	Ischaemic stroke / SE<br>All-cause mortality<br>Any bleeding<br>Intracranial / gastrointestinal bleeding	1 year	=
Lip, 2018, USA 77<br><br>Retrospective with PSM	100,977 AF patients on Apixaban<br>Mean age 76.1<br>49% Female<br>Mean CHA_2DS_2-VASC 3.9	100,977 AF patients on Warfarin<br>Mean age 76.0<br>49% Female<br>Mean CHA_2DS_2-VASC 3.9	Stroke / SE<br>Ischaemic stroke<br>Haemorrhagic stroke<br>SE<br>Major bleeding	Mean follow-up in days<br><br>Apixaban vs Warfarin:<br>Apixaban = 187.6<br>Warfarin = 242.3	+

First author, Year, country<br><br>Study design	Apixaban<br>*n*=participants<br>mean (±SD) Age (years)<br>% Female/Male<br>Charlson comorbidity index<br>CHA_2DS_2-VASc score<br>HAS-BLED score	Comparator(s)<br>*n*=patients<br>mean (±SD) Age (years)<br>% Female/Male<br>Charlson comorbidity index<br>CHA_2DS_2-VASc score<br>HAS-BLED score	Outcomes	Follow-up	Impact of Apixaban<br><br>+ Favours Apixaban<br>- Favours comparator<br>= No difference / uncertain
	Mean HAS-BLED 3.1<br><br>37,314 AF patients on Apixaban<br>Mean age 73.2<br>Mean CHA_2DS_2-VASC 3.5<br>Mean HAS-BLED 2.8<br><br>107,236 AF patients on Apixaban<br>Mean age 75.2<br>48% Female<br>Mean CHA_2DS_2-VASC 3.8<br>Mean HAS-BLED 3.0	Mean HAS-BLED 3.0<br><br>37,314 AF patients on Dabigatran<br>Mean age 73.0<br>Mean CHA_2DS_2-VASC 3.5<br>Mean HAS-BLED 2.8<br><br>107,236 AF patients on Rivaroxaban<br>Mean age 75.1<br>48.5% Female<br>Mean CHA_2DS_2-VASC 3.7<br>Mean HAS-BLED 3.0	GI bleeding<br>Intracranial haemorrhage<br>Other bleeding	 Apixaban vs Dabigatran<br>Apixaban = 186.0<br>Dabigatran = 226.3<br><br>Apixaban vs Rivaroxaban<br>Apixaban = 187.2<br>Rivaroxaban = 230.3	
Lip, 2020, USA 78<br><br>Retrospective with PSM	41,662 AF patients on Apixaban<br>Mean age 76.9 (±8.9)<br>51% Female<br>Mean CHA_2DS_2-VASC 4.4 (±1.6)<br>Mean HAS-BLED 3.5 (±1.3)<br>Mean Charlson 3.9 (±2.8)<br><br>13,969 AF patients on Apixaban<br>Mean age 74.6 (±9.2)<br>48.6% Female<br>Mean CHA_2DS_2-VASC 4.1 (±1.6)<br>Mean HAS-BLED 3.2 (±1.3)<br>Mean Charlson 3.4 (±2.7)<br><br>43,250 AF patients on Apixaban	41,662 AF patients on Warfarin<br>Mean age 76.9 (±8.8)<br>52% Female<br>Mean CHA_2DS_2-VASC 4.4 (±1.5)<br>Mean HAS-BLED 3.5 (±1.3)<br>Mean Charlson 3.9 (±2.8)<br><br>13,969 AF patients on Dabigatran<br>Mean age 74.6 (±9.1)<br>48% Female<br>Mean CHA_2DS_2-VASC 4.1 (±1.6)<br>Mean HAS-BLED 3.2 (±1.3)<br>Mean Charlson 3.5 (±2.7)	Stroke / SE<br>Ischaemic stroke<br>Haemorrhagic stroke<br>SE<br>Major bleeding<br>GI bleeding<br>Intracranial haemorrhage<br>Other bleeding	Mean follow-up in days<br><br>Apixaban vs Warfarin:<br>Apixaban = 186.8<br>Warfarin = 238.4<br><br>Apixaban vs Dabigatran<br>Apixaban = 191.0<br>Dabigatran = 233.6<br><br>Apixaban vs Rivaroxaban<br>Apixaban = 187.7<br>Rivaroxaban = 229.9	+

First author, Year, country<br><br>**Study design**	**Apixaban**<br>*n*=participants<br>mean (±SD) Age (years)<br>% Female/Male<br>Charlson comorbidity index<br>CHA_2DS_2-VASc score<br>HAS-BLED score	**Comparator(s)**<br>*n*=patients<br>mean (±SD) Age (years)<br>% Female/Male<br>Charlson comorbidity index<br>CHA_2DS_2-VASc score<br>HAS-BLED score	**Outcomes**	**Follow-up**	**Impact of Apixaban**<br><br>+ Favours Apixaban<br>- Favours comparator<br>= No difference / uncertain
	Mean age 76.2 (±9.2)<br>51.1% Female<br>Mean CHA_2DS_2-VASC 4.3 (±1.6)<br>Mean HAS-BLED 3.4 (±1.3)<br>Mean Charlson 3.7 (±2.8)	43,250 AF patients on Rivaroxaban<br>Mean age 76.1 (±9.2)<br>51% Female<br>Mean CHA_2DS_2-VASC 4.3 (±1.6)<br>Mean HAS-BLED 3.4 (±1.3)<br>Mean Charlson 3.7 (±2.8)			
Martinez, 2018, USA 20<br><br>Retrospective with PSM	Propensity matched cohort:<br><br>1,392 AF patients on Apixaban<br>Median age 86 (IQR 83-89)<br>64% Male<br>Median CHA_2DS_2-VASC 4 (IQR 4-5)<br>Median HAS-BLED 2 (IQR 2-3)	Propensity matched cohort:<br><br>1,392 AF patients on Warfarin<br>Median age 86 (IQR 83-89)<br>63% Male<br>Median CHA_2DS_2-VASC 4 (IQR 4-5)<br>Median HAS-BLED 2 (2-3)	Stroke / SE<br>Ischaemic stroke<br>Major bleed<br>Haemorrhagic stroke<br>Intracranial haemorrhage<br>GI bleed	Median 0.9 (IQR 0.4-1.6) years<br><br>1 year and 2 year follow-up also provided given in results	-
Mentias, 2020, USA 79<br><br>Retrospective with PSM	Propensity matched cohorts based on degree of polypharmacy<br><br>High polypharmacy (>8 meds):<br>723 AF patients on Apixaban<br>Mean age 74.8 (±6.6)<br>53% Female<br>Mean CHA_2DS_2-VASC 5.1 (±1.6)<br>Mean HAS-BLED 3.13 (±1.0)<br><br>Medium polypharmacy (4-8 meds):<br>1,487 AF patients on Apixaban	Propensity matched cohorts based on degree of polypharmacy<br><br>High polypharmacy (>8 meds):<br>723 AF patients on Rivaroxaban<br>Mean age 74.5 (±6.5)<br>51% Female<br>Mean CHA_2DS_2-VASC 5.0 (±1.7)<br>Mean HAS-BLED 3.2 (±1.1)<br><br>723 AF patients on Warfarin<br>Mean age 74.4 (±6.6)<br>54% Female	Ischaemic stroke<br>Bleeding<br>GI bleeding<br>Intracranial bleeding<br>All-cause mortality	Not clearly stated - they reported crude rates as per 100 patient years but the paper does not appear to clarify average follow-up duration per drug	=

First author, Year, country<br><br>**Study design**	**Apixaban**<br>*n*=participants<br>mean (±SD) Age (years)<br>% Female/Male<br>Charlson comorbidity index<br>CHA_2DS_2-VASc score<br>HAS-BLED score	**Comparator(s)**<br>*n*=patients<br>mean (±SD) Age (years)<br>% Female/Male<br>Charlson comorbidity index<br>CHA_2DS_2-VASc score<br>HAS-BLED score	**Outcomes**	**Follow-up**	**Impact of Apixaban**<br><br>+ Favours Apixaban<br>- Favours comparator<br>= No difference / uncertain
	Mean age 75.3 (±6.4)<br>50% Female<br>Mean CHA_2DS_2-VASC 4.5 (±1.6)<br>Mean HAS-BLED 2.8 (±1.0)<br><br>Low polypharmacy (<=3 meds):<br>1,219 AF patients on Apixaban<br>Mean age 76.0 (±6.4)<br>47% Female<br>Mean CHA_2DS_2-VASC 4.2 (±1.6)<br>Mean HAS-BLED 2.5 (±0.9)	Mean CHA_2DS_2-VASC 5.1 (±1.7)<br>Mean HAS-BLED 3.1 (±1.1)<br><br>Medium polypharmacy (4-8 meds):<br>1,487 AF patients on Rivaroxaban<br>Mean age 75.2 (±6.5)<br>48% Female<br>Mean CHA_2DS_2-VASC 4.5 (±1.6)<br>Mean HAS-BLED 2.7 (±1.0)<br><br>1,487 AF patients on Warfarin<br>Mean age 75.3 (±6.7)<br>50% Female<br>Mean CHA_2DS_2-VASC 4.6 (±1.6)<br>Mean HAS-BLED 2.8 (±1.0)<br><br>Low polypharmacy (<=3 meds):<br>1,219 AF patients on Rivaroxaban<br>Mean age 75.7 ± 6.4<br>46% Female<br>Mean CHA_2DS_2-VASC 4.14 ± 1.60<br>Mean HAS-BLED 2.47 ± 0.91<br><br>1,219 AF patients on Warfarin<br>Mean age 76.0 ± 6.6			

**First author, Year, country**<br><br>**Study design**	**Apixaban**<br>*n*=participants<br>mean (±SD) Age (years)<br>% Female/Male<br>Charlson comorbidity index<br>CHA_2DS_2-VASc score<br>HAS-BLED score	**Comparator(s)**<br>*n*=patients<br>mean (±SD) Age (years)<br>% Female/Male<br>Charlson comorbidity index<br>CHA_2DS_2-VASc score<br>HAS-BLED score	**Outcomes**	**Follow-up**	**Impact of Apixaban**<br><br>+ Favours Apixaban<br>- Favours comparator<br>= No difference / uncertain
		47% Female<br>Mean CHA_2DS_2-VASC 4.25 ± 1.54<br>Mean HAS-BLED 2.49 ± 0.92			
Mitsuntisuk, 2021, Thailand 34<br><br>Retrospective with PSM	Unadjusted cohort (PSM used but matched cohort data not given)<br><br>405 AF patients on Apixaban<br>Mean age 73.9 (±10.2)<br>50% Female<br>Mean CHA_2DS_2-VASC 3.9 (±1.7)<br>Mean HAS-BLED 1.7 (±1.0)	Unadjusted cohort (PSM used but matched cohort data not given)<br><br>605 AF patients on Warfarin<br>Mean age 68.4 (±11.4)<br>50% Female<br>Mean CHA_2DS_2-VASC 3.3 (±1.8)<br>Mean HAS-BLED 1.3 (±0.9)	Stroke / SE<br>Ischaemic stroke<br>Haemorrhagic stroke<br>Major bleeding<br>Intracranial haemorrhage<br>GI bleeding<br>Other sites major bleeding	Apixaban = 1.90 years<br>Warfarin = 2.82 years<br><br>Followed until first occurrence of any outcome or end of study period.	+
Mueller, 2019, UK 46<br><br>Retrospective with adjusted HRs	6,200 AF patients on Apixaban<br>Mean age 73.7 (±11.5)<br>47% Female<br>Mean CHA_2DS_2-VASC 2.9 (±1.7)<br>Mean HAS-BLED 2.1 (±1.2)<br>Mean Charlson 1.4 (±1.7)	1,112 AF patients on Dabigatran<br>Mean age 71.1 (±12.0)<br>37% Female<br>Mean CHA_2DS_2-VASC 2.5 (±1.8)<br>Mean HAS-BLED 1.9 (±1.2)<br>Mean Charlson 1.1 ±1.5)<br><br>7,265 AF patients on Rivaroxaban<br>Mean age 74.8 (±11.0)<br>46% Female<br>Mean CHA_2DS_2-VASC 3.0 (±1.7)<br>Mean HAS-BLED 2.0 (±1.2)<br>Mean Charlson 1.3 (±1.7)	Ischaemic stroke<br>SE<br>Cardiovascular death<br>Pulmonary embolism<br>TIA<br>Myocardial infarction<br>All-cause mortality<br>Haemorrhagic stroke<br>GI bleeding<br>Other major bleeds	Apixaban = 188 days<br>Dabigatran = 216 days	=
Nielsen, 2017, Denmark 35	Unadjusted cohorts<br>4,400 patients on Apixaban	Unadjusted cohort<br>38,893 patients on Warfarin	Ischaemic stroke / SE<br>Ischaemic stroke	1 year + 2.5 year	=

First author, Year, country<br><br>Study design	Apixaban<br>*n*=participants<br>mean (±SD) Age (years)<br>% Female/Male<br>Charlson comorbidity index<br>CHA_2DS_2-VASc score<br>HAS-BLED score	Comparator(s)<br>*n*=patients<br>mean (±SD) Age (years)<br>% Female/Male<br>Charlson comorbidity index<br>CHA_2DS_2-VASc score<br>HAS-BLED score	Outcomes	Follow-up	Impact of Apixaban<br><br>+ Favours Apixaban<br>- Favours comparator<br>= No difference / uncertain
Retrospective with Inverse Probability of Treatment Weighting	Mean age 83.9 (±8.2)<br>61% Female<br>Mean CHA_2DS_2-VASC 4.3 (±1.5)<br>Mean HAS-BLED 2.8 (±1.5)	Mean age 71.0 (±12.6)<br>40% Female<br>Mean CHA_2DS_2-VASC 3.0 (±1.7)<br>Mean HAS-BLED 2.4 (±1.2)	All-cause mortality<br>Any bleeding<br>Major bleeding<br>Haemorrhagic stroke	Mean follows ups not given but states overall mean was 2.3 years with shortest mean in Apixaban group of 1 year.	
Noseworthy, 2016, USA 80<br><br>Retrospective with PSM	Propensity matched cohorts:<br>Apixaban vs Dabigatran:<br>6,542 AF patients on Apixaban<br>Median age 73 (IQR 65-81)<br>54% Male<br>Median CHA_2DS_2-VASC 4 (IQR 3-5)<br>Median HAS-BLED 2 (IQR 2-3)<br>Median Charlson 2 (IQR 1-4)<br><br>Apixaban vs Rivaroxban:<br>6,565 AF patients on Apixaban<br>Median age 73 (IQR 65-81)<br>54% Male<br>Median CHA_2DS_2-VASC 4 (IQR 3-5)<br>Median HAS-BLED 2 (IQR 2-3)<br>Median Charlson 2 (IQR 1-4)	Propensity matched cohorts:<br>Apixaban vs Dabigatran:<br>6,542 AF patients on Dabigatran<br>Median age 73 (IQR 65-81)<br>54% Male<br>Median CHA_2DS_2-VASC 4 (IQR 3-5)<br>Median HAS-BLED 2 (IQR 2-3)<br>Median Charlson 2 (IQR 1-4)<br><br>Apixaban vs Rivaroxban:<br>6,565 AF patients on Rivaroxaban<br>Median age 73 (IQR 65-81)<br>54% Male<br>Median CHA_2DS_2-VASC 4 (IQR 3-5)<br>Median HAS-BLED 2 (IQR 2-3)<br>Median Charlson 2 (IQR 1-4)	Stroke / SE<br>Ischaemic stroke<br>Haemorrhagic stroke<br>Major bleeding<br>Intracranial bleeding	Follow-up per cohort NR.<br><br>Results presented as event rates /100 PY.<br><br>Sensitivity analysis by censoring at 6 months was reportedly not significantly different to overall outcome.	+
Noseworthy, 2017, USA 22<br><br>Retrospective	12,949 AF patients on Apixaban<br>Median age 74 (IQR 67 - 81)<br>48% Female<br>CHA_2DS_2-VASc </= 3 42%<br>CHA_2DS_2-VASc 4 22%	68,804 AF patients on Warfarin<br>Median age 73 (IQR 64-80)<br>42.7% Female<br>CHA_2DS_2-VASc </= 3 41.4%<br>CHA_2DS_2-VASc 4 21.1%	Stroke<br>Major bleeding	12-months	=

First author, Year, country<br><br>Study design	Apixaban<br>*n*=participants<br>mean (±SD) Age (years)<br>% Female/Male<br>Charlson comorbidity index<br>CHA_2DS_2-VASc score<br>HAS-BLED score	Comparator(s)<br>*n*=patients<br>mean (±SD) Age (years)<br>% Female/Male<br>Charlson comorbidity index<br>CHA_2DS_2-VASc score<br>HAS-BLED score	Outcomes	Follow-up	Impact of Apixaban<br><br>+ Favours Apixaban<br>- Favours comparator<br>= No difference / uncertain
	CHA_2DS_2-VASc >/= 5 35.9%<br><br>HAS-BLED 0,1 19.5%<br>HAS-BLED 2 36.3%<br>HAS-BLED 3 27.3%<br>HAS-BLED >/=4 16.8%	CHA_2DS_2-VASc >/= 5 37.4%<br><br>HAS-BLED 0,1 21.5%<br>HAS-BLED 2 34.2%<br>HAS-BLED 3 27.3%<br>HAS-BLED >/=4 17.1%<br><br>9,412 AF patients on Dabigatran<br>Median age 69 (IQR 61-77)<br>38.7% Female<br>CHA_2DS_2-VASc </= 3 56.4%<br>CHA_2DS_2-VASc 4 19.6%<br>CHA_2DS_2-VASc >/= 5 23.9%<br><br>HAS-BLED 0,1 31.6%<br>HAS-BLED 2 36.3%<br>HAS-BLED 3 21.9%<br>HAS-BLED >/=4 10.2%<br><br>68,804 AF patients on Rivaroxaban<br>Median age 71 (IQR 63-78)<br>41.2% Female<br>CHA_2DS_2-VASc </= 3 52.4%<br>CHA_2DS_2-VASc 4 20.6%<br>CHA_2DS_2-VASc >/= 5 27%<br><br>HAS-BLED 0,1 26.8%<br>HAS-BLED 2 37.1%			

First author, Year, country<br><br>**Study design**	**Apixaban**<br>*n*=participants<br>mean (±SD) Age (years)<br>% Female/Male<br>Charlson comorbidity index<br>CHA_2DS_2-VASc score<br>HAS-BLED score	**Comparator(s)**<br>*n*=patients<br>mean (±SD) Age (years)<br>% Female/Male<br>Charlson comorbidity index<br>CHA_2DS_2-VASc score<br>HAS-BLED score	**Outcomes**	**Follow-up**	**Impact of Apixaban**<br><br>+ Favours Apixaban<br>- Favours comparator<br>= No difference / uncertain
		HAS-BLED 3 24.5%<br>HAS-BLED >/=4 11.6%			
Ogawa, 2011, Japan 14<br><br>RCT	74 patients on Apixaban (2.5mg BD)<br>- 72 treated, 7 discontinued<br>Mean age 69<br>Male 85%<br><br>74 patients on Apixaban (5mg BD)<br>- 71 treated, 5 discontinued<br>Mean age 70<br>Male 82%	74 patients Warfarin as per INR<br>Mean age 72<br>Male 81%	Composite primary outcome was major and clinically relevant non-major (CRNM) bleeding.<br><br>Secondary endpoints: major or CRNM bleeding separately, total bleeding composite, minor bleeding.<br><br>Bleeding defined using ISTH criteria.<br><br>Efficacy endpoints: composite of stroke and SE; composite of stroke SE or all-cause death; composite of MI or all-cause death.	12-weeks	=
Perreault, 2021, Canada 47<br><br>Retrospective with PSM	6,771 patients on Apixaban<br>Mean age 76.3 (±8.5)<br>51% Male<br>Mean CHA_2DS_2-VASc 3.4 (±1.4)<br>Mean HAS-BLED 2.9 (±1.3)<br>Mean Charlson 4.4 (±3.4)	4,632 patients on Rivaroxaban<br>Age mean 73.2 (±9.1)<br>Male 55%<br>Mean CHA_2DS_2-VASc 3.0 (±1.4)<br>Mean HAS-BLED 2.5 (±1.3)<br>Mean Charlson 3.6 (±3.2)	Primary:<br>(i) Composite of ischaemic stroke/SE, acute MI, all-cause mortality.<br>(ii) composite of major	12-months	+

**First author, Year, country**<br><br>**Study design**	**Apixaban**<br>*n*=participants<br>mean (±SD) Age (years)<br>% Female/Male<br>Charlson comorbidity index<br>CHA_2DS_2-VASc score<br>HAS-BLED score	**Comparator(s)**<br>*n*=patients<br>mean (±SD) Age (years)<br>% Female/Male<br>Charlson comorbidity index<br>CHA_2DS_2-VASc score<br>HAS-BLED score	**Outcomes**	**Follow-up**	**Impact of Apixaban**<br><br>+ Favours Apixaban<br>- Favours comparator<br>= No difference / uncertain
			bleeding (ICH, GI and major from other sites).<br><br>Secondary:<br>Stroke/SE/death<br>Stroke/SE<br>Death<br>Acute MI<br>Bleeding outcomes		
Ramagopalan, 2019, Spain 36<br><br>Retrospective with PSM	2,160 Apixaban<br>Age mean 71.2 (±12.8)<br>48% Male<br>Mean Charlson 2.5 (±2.0)<br>Mean CHA_2DS_2-VASc 3.3 (±1.9)<br>Mean HAS-BLED 2.0 (±1.3)	2,160 patients on VKA (acenocoumarol or warfarin)<br>Mean age 71.6 (±10.1)<br>48% Male<br>Mean Charlson 2.6 (±1.9)<br>Mean CHA_2DS_2-VASc mean 3.4 (±1.7)<br>Mean HAS-BLED 2.1 (±1.1)	Ischaemic stroke and SE<br>Major bleeding (intracranial, GI, other sites requiring hospital admission)<br>Minor bleeding	12-months	+
Ramagopalan, 2018, Spain 81<br><br>Retrospective with PSM	1,521 patients on Apixaban<br>Median age 79 (IQR 35-100)<br>49% Male	Pre-PSM<br>8,393 patients on VKA<br>Median age 78 (IQR 20-104)<br>52% Male	Major bleeding (including GI bleeding, intracranial bleeding, bleeding from other sites).	10-months	+
Ray, 2021, USA 18<br><br>Retrospective with Inverse Probability of Treatment Weighting	353,879 patients on Apixaban<br>Mean age 77 (±7)<br>50% Male<br>Mean CHA_2DS_2-VASc 4.3	227,572 patients on Rivaroxaban<br>Mean age 77 (±7)<br>50% Male<br>Mean CHA_2DS_2-VASc 4.3	Major ischaemic or haemorrhagic events<br>Mortality<br>Ischaemic stroke<br>ICH	4-years	+

**First author, Year, country**<br><br>**Study design**	**Apixaban**<br>*n*=participants<br>mean (±SD) Age (years)<br>% Female/Male<br>Charlson comorbidity index<br>CHA_2DS_2-VASc score<br>HAS-BLED score	**Comparator(s)**<br>*n*=patients<br>mean (±SD) Age (years)<br>% Female/Male<br>Charlson comorbidity index<br>CHA_2DS_2-VASc score<br>HAS-BLED score	**Outcomes**	**Follow-up**	**Impact of Apixaban**<br><br>+ Favours Apixaban<br>- Favours comparator<br>= No difference / uncertain
Rodríguez-Bernal, 2018, Spain 44<br><br>Retrospective with Inverse Probability of Treatment Weighting	32,476 patients on Acenocoumarol<br>Mean age 74.8 (±9.6)<br>48% Female<br>Mean CHA_2DS_2-VASc 3.8 (±1.65)<br>Mean HAS-BLED 2.9 (±1.2)	2,259 patients on Apixaban<br>Mean age 75.0 (±10.7)<br>48% Female<br>Mean CHA_2DS_2-VASc 3.9 (±1.8)<br>Mean HAS-BLED 3.0 (±1.3)	Mortality<br>Ischaemic stroke<br>TIA<br>GI bleed<br>ICH	Average follow-up of 1.8 years	=
Rutherford, 2020, Norway 40<br><br>Retrospective with PSM	Dabigatran-apixaban PSM<br>10,413 patients on Apixaban<br>Mean age 70.6 (±11.7)<br>62% Male<br>Mean CHA_2DS_2-VASc 2.94 (±1.72)<br>Mean HAS-BLED 2.3 (±1.2)<br><br>Apixaban-rivaroxaban PSM<br>13,699 patients on Apixaban<br>Age 72.7 (±11.7)<br>58% Male<br>Mean CHA_2DS_2-VASc 3.2 (±1.7)<br>Mean HAS-BLED 2.4 (±1.2)	Dabigatran-apixaban PSM<br>10,413 patients on Dabigatran<br>Age 70.6 (±11.2)<br>62% Male<br>Mean CHA_2DS_2-VASc 2.7 (±1.7)<br>Mean HAS-BLED 2.3 (±1.2)<br><br>Apixaban-rivaroxaban PSM<br>13,699 patients on Rivaroxaban<br>Mean age 72.7 (±11.1)<br>58% Male<br>Mean CHA_2DS_2-VASc 3.2 (±1.7)<br>Mean HAS-BLED 2.4 (±1.1)	Stroke<br>Embolism,<br>time to first ischaemic stroke.<br><br>Time to first major bleed, clinically relevant non-major bleeding (CRNM), major or CRNM bleeding, GI bleeding, intracranial haemorrhage.	12 months	+
Själander, 2018, Sweden 82<br><br>Retrospective with Full Optimal Matching	12,311 patients on Apixaban<br>Age 75 (78-82)<br>55% Male<br>Mean CHA_2DS_2-VASc 3.2 (±1.8)	37,174 patients on Warfarin<br>Age 75 (68-81)<br>60% Male<br>Mean CHA_2DS_2-VASc 3.2 (±1.8)	Stroke and SE<br>stroke<br>Ischaemic stroke<br>Haemorrhagic stroke (ICH or SAH)<br><br>Major bleed (ICH, GI, fatal or requiring	Study length NR.<br><br>37,174 patients (56,164 treatment years) were on warfarin	+

**First author, Year, country**<br><br>**Study design**	**Apixaban**<br>*n*=participants<br>mean (±SD) Age (years)<br>% Female/Male<br>Charlson comorbidity index<br>CHA_2DS_2-VASc score<br>HAS-BLED score	**Comparator(s)**<br>*n*=patients<br>mean (±SD) Age (years)<br>% Female/Male<br>Charlson comorbidity index<br>CHA_2DS_2-VASc score<br>HAS-BLED score	**Outcomes**	**Follow-up**	**Impact of Apixaban**<br><br>+ Favours Apixaban<br>- Favours comparator<br>= No difference / uncertain
			hospital care)<br>All-cause mortality	6574 patients (8326 treatment years) on dabigatran,<br><br>8323 patients (7956 treatment years) rivaroxaban<br><br>12,311 patients (8730 treatment years) on apixaban.	
Staerk, 2017, Denmark 37<br><br>Retrospective with adjusted HRs	6,899 patients on Apixaban<br>Age 76 (68-84)<br>50% Male<br>CHA_2DS_2-VASc 3.1 (±1.6)<br>HAS-BLED 2.2 (±1.2)	18,094 patients on VKA<br>Age 73 (65-80)<br>58% Male<br>CHA_2DS_2-VASc 2.9 (±1.6)<br>HAS-BLED 2.2 (±1.2)	Stroke/SE<br>Intracranial bleeding (ICH, SAH, traumatic subdural, traumatic epidural)	Study period = 2 years.<br><br>Drug specific follow up duration (medians)<br>VKA = 252 days<br>Dabigatran = 386 days<br>Rivaroxaban = 208 days<br>Apixaban = 204 days	+
Staerk, 2018, Denmark 41<br><br>Retrospective with adjusted HRs	7,203 patients on Apixaban<br>58% Male<br>Median age 71 (65, 77)<br>Median CHA_2DS_2-VASc 3 (2, 4)<br>Median HAS-BLED 2 (1, 3)	7,078 patients on Dabigatran<br>64% Male<br>Median age 67 (61, 71)<br>Median CHA_2DS_2-VASc 2 (1, 3)<br>Median HAS-BLED 2 (1, 2)<br><br>6,868 patients on Rivaroxaban<br>55% Male<br>Median age 71 (65, 78)	Stroke/SE<br>Ischaemic stroke<br>Major bleeding<br>Intracranial bleeding<br>GI bleeding	Study period = 2 years.<br><br>Average duration of each cohort follow-up NR.	=

First author, Year, country<br><br>Study design	Apixaban<br>*n*=participants<br>mean (±SD) Age (years)<br>% Female/Male<br>Charlson comorbidity index<br>CHA_2DS_2-VASc score<br>HAS-BLED score	Comparator(s)<br>*n*=patients<br>mean (±SD) Age (years)<br>% Female/Male<br>Charlson comorbidity index<br>CHA_2DS_2-VASc score<br>HAS-BLED score	Outcomes	Follow-up	Impact of Apixaban<br><br>+ Favours Apixaban<br>- Favours comparator<br>= No difference / uncertain
		Median CHA_2DS_2-VASc 3 (2,4)<br>Median HAS-BLED 2 (1,3)			
Tepper, 2018, USA 50<br><br>Retrospective with adjusted HRs	8,785 patients on Apixaban<br>Mean age 70 (±12)<br>37% Female<br>Mean Charlson = 1.8 (±2)<br>Mean CHA_2DS_2-VASc 2.5 (±1.5)<br>Mean HAS-BLED 1.9 (±1.2)	20,963 patients on Dabigatran<br>Mean age 71 (±11)<br>35% Female<br>Mean Charlson 1.6 (±1.9)<br>Mean CHA_2DS_2-VASc 2.4 (±1.4)<br>Mean HAS-BLED 1.8 (±1.2)<br><br>30,529 patients on Rivaroxaban<br>Mean age = 68 (±12)<br>37% Female<br>Mean Charlson 1.8 (±2.2)<br>Mean CHA_2DS_2-VASc 2.4 (±1.5)<br>Mean HAS-BLED 1.8 (±1.2)	Any bleeding<br>CRNM bleeding<br>Inpatient major bleeding total<br>Intracranial Haemorrhage<br>GI bleeding<br>Other	Apixaban = 184 days<br>Dabigatran = 553 days<br>Rivaroxaban = 300 days	+
Tiew, 2020, Singapore 17<br><br>Retrospective with adjusted HRs	98 patients on Apixaban<br>Mean age 72.9 (±10.6)<br>46% Female<br>Median CHA_2DS_2-VASc 3 (2-4)<br>Median HAS-BLED 1 (1-2)	157 patients on Warfarin<br>Mean age 70.4 (±10.8)<br>66% Female<br>Median CHA_2DS_2-VASc 3 (2-4)<br>Median HAS-BLED 1 (1-2)<br><br>154 patients on Rivaroxaban<br>Mean age 70.5 (±11.1)<br>66% Female<br>Median CHA_2DS_2-VASc 3 (2-4)<br>Median HAS-BLED 1 (1-2)<br><br>30 patients on Dabigatran	Primary:<br>Major bleeding and stroke.<br><br>Secondary:<br>Overall bleeding (major bleeding and clinically relevant non-major bleeding)<br>Thromboembolic events (stroke, TIA and SE).	Warfarin = 691.7 days<br>Rivaroxaban = 690.5 days<br>Apixaban = 684.5 days<br>Dabigatran = 609.8 days	=

**First author, Year, country**<br><br>**Study design**	**Apixaban**<br>*n*=participants<br>mean (±SD) Age (years)<br>% Female/Male<br>Charlson comorbidity index<br>CHA_2DS_2-VASc score<br>HAS-BLED score	**Comparator(s)**<br>*n*=patients<br>mean (±SD) Age (years)<br>% Female/Male<br>Charlson comorbidity index<br>CHA_2DS_2-VASc score<br>HAS-BLED score	**Outcomes**	**Follow-up**	**Impact of Apixaban**<br><br>+ Favours Apixaban<br>- Favours comparator<br>= No difference / uncertain
		Mean age 67.4 (±11.4)<br>13% Female<br>Median CHA_2DS_2-VASc 2 (1-4)<br>Median HAS-BLED 1 (1-2)			
Van Ganse, 2020, France 83<br><br>Retrospective with PSM and adjusted effect measures	87,565 AF patients on Apixaban<br>Mean age 74.7 (±11.5)<br>51% Male<br>Mean CHA_2DS_2-VASC 3.1 (±1.7)<br>Mean HAS-BLED 2.2 (±1.0)<br>Mean Charlson 4.5 (±2.3)	112,628 AF patients on VKA<br>Mean age 78.5 (±11.1)<br>49% Male<br>Mean CHA_2DS_2-VASC 3.9 (±1.7)<br>Mean HAS-BLED 2.6 (±1.1)<br>Mean Charlson 5.9 (±2.6)<br><br>100,063 AF patients on Rivaroxaban<br>Mean age 72.0 (±12.0)<br>55% Male<br>Mean CHA_2DS_2-VASC 2.7 (±1.7)<br>Mean HAS-BLED 2.0 (±1.0)<br>Mean Charlson 4.0 (±2.2)<br><br>21,245 AF patients on Dabigatran<br>Mean age 72.7 (±11.8)<br>54% Male<br>Mean CHA_2DS_2-VASC 2.8 (±1.7)<br>Mean HAS-BLED 2.1 (±1.0)<br>Mean Charlson 4.1 (±2.2)	All-cause mortality<br>Stroke/SE<br>Major bleeding	Mean follow-up duration was 316 days<br><br>For those receiving apixaban, rivaroxaban, and dabigatran, the mean follow-up duration was 286, 318, and 329 days, respectively.	+
Villines, 2019, USA 51	4,802 AF patients on Apixaban<br>Mean age 70.2 (±10.0)<br>63% Male	4,802 AF patients on Dabigatran<br>Mean age 70.2 (±10.2)<br>63% Male	Stroke<br>Major bleeding	Dabigatran = 349.5 days<br>Apixaban = 357.7 days	=

First author, Year, country<br><br>**Study design**	**Apixaban**<br>*n*=participants<br>mean (±SD) Age (years)<br>% Female/Male<br>Charlson comorbidity index<br>CHA_2DS_2-VASc score<br>HAS-BLED score	**Comparator(s)**<br>*n*=patients<br>mean (±SD) Age (years)<br>% Female/Male<br>Charlson comorbidity index<br>CHA_2DS_2-VASc score<br>HAS-BLED score	**Outcomes**	**Follow-up**	**Impact of Apixaban**<br><br>+ Favours Apixaban<br>- Favours comparator<br>= No difference / uncertain
Retrospective with PSM	Mean CHA_2DS_2-VASC 3.0 (±1.6)<br>Mean HAS-BLED 2.3 (±1.2)<br>Mean Charlson 4.2 (±2.4)	Mean CHA_2DS_2-VASC 3.0 (±1.7)<br>Mean HAS-BLED 2.3 (±1.2)<br>Mean Charlson 4.2 (±2.5)			
Vinogradova, 2018, UK (Qresearch Database) 16<br><br>Prospective Cohort	9,199 AF patients on Apixaban<br>76.5 (10.9)<br>52% Male	53,921 AF patients on Warfarin<br>74.8 (10.4)<br>56% Male<br><br>4,534 AF patients on Dabigatran<br>74.7 (10.7)<br>58% Male<br><br>13,597 AF patients on Rivaroxaban<br>75.8 (10.9)<br>54% Male	All-cause mortality<br>Ischaemic stroke<br>Major bleeding<br>GI bleed<br>Intracranial bleed	Median Days of treatment<br><br>Apixaban 248<br>Warfarin 344<br>Dabigatran 271<br>Rivaroxaban 265	+
Vinogradova, 2018, UK (CPRD Database) 16<br><br>Prospective with adjusted effect measures	1,402 AF patients on Apixaban<br>76.6 (10.9)<br>55% Male	16,664 AF patients on Wafarin<br>74.8 (10.3)<br>56% Male<br><br>1,003 AF patients on Dabigatran<br>74.4 (10.8)<br>62% Male<br><br>2,950 AF patients on Rivaroxaban<br>75.9 (10.8)<br>54% Male	All-cause mortality<br>Ischaemic stroke<br>Major bleeding<br>GI bleed<br>Intracranial bleed	Median Days of treatment<br><br>Apixaban 143<br>Warfarin 286<br>Dabigatran 214<br>Rivaroxaban 163	+
Wanat, 2019, USA 38	10,189 AF patients on Apixaban<br>Mean age 72.1 (±9.1)	10,189 AF patients on Warfarin<br>Mean age 72.1 (±9.2)	Stroke/SE<br>Major bleed	12 months	+

**First author, Year, country**<br><br>**Study design**	**Apixaban**<br>*n*=participants<br>mean (±SD) Age (years)<br>% Female/Male<br>Charlson comorbidity index<br>CHA_2DS_2-VASc score<br>HAS-BLED score	**Comparator(s)**<br>*n*=patients<br>mean (±SD) Age (years)<br>% Female/Male<br>Charlson comorbidity index<br>CHA_2DS_2-VASc score<br>HAS-BLED score	**Outcomes**	**Follow-up**	**Impact of Apixaban**<br><br>+ Favours Apixaban<br>- Favours comparator<br>= No difference / uncertain
Retrospective with PSM	53% Male<br>Mean CHA_2DS_2-VASC 2.5 (±1.3)<br>Mean HAS-BLED 1.5 (±0.9)	53% Male<br>Mean CHA_2DS_2-VASC 2.4 (±1.3)<br>Mean HAS-BLED 1.5 (±0.9)			
Yang, 2020, USA 54<br><br>Retrospective with adjusted HRs	494 post-stroke AF patients on Apixaban<br>Mean age 80.3 (8.3)<br>35% Male<br>Mean CHA_2DS_2-VASC 6.8 (±1.3)<br>Mean HAS-BLED 4.7 (±0.8)	247 post-stroke AF patients on Dabigatran<br>Mean age 78.3 (8.4)<br>37% Male<br>Mean CHA_2DS_2-VASC 6.6 (±1.3)<br>Mean HAS-BLED 4.6 (±0.8)<br><br>1,104 post-stroke AF patients on Rivaroxaban<br>Mean age 78.8 (8.6)<br>44% Male<br>Mean CHA_2DS_2-VASC 6.7 (±1.3)<br>Mean HAS-BLED 4.7 (±0.8)<br><br>3,082 post-stroke AF patients on Warfarin<br>Mean age 77.9 (10.0)<br>39% Male<br>Mean CHA_2DS_2-VASC 6.8 (±1.3)<br>Mean HAS-BLED 4.7 (±0.8)	Ischaemic stroke<br>Any bleeding event<br>GI bleeding	Apixaban 178 days<br>Dabigatran 271 days<br>Rivaroxaban 256 days<br>Warfarin 267 days	-
Yao, 2016, USA 84<br><br>Retrospective with PSM	7,695 AF patients on Apixaban<br>Median age 73 (IQR 66-81)<br>47% Female<br>Median CHA_2DS_2-VASC 4 (IQR 3-5)<br>Median HAS-BLED 2 (IQR 2-3)	7,695 AF patients on Warfarin<br>Median age 73 (IQR 66-81)<br>47% Female<br>Median CHA_2DS_2-VASC 4 (IQR 3-5)<br>Median HAS-BLED 2 (IQR 2-3)	Stroke/SE<br>Ischaemic stroke<br>Haemorrhagic stroke<br>Major bleeding<br>ICH<br>GI bleed	6-months	+

**First author, Year, country**<br><br>**Study design**	**Apixaban**<br>*n*=participants<br>mean (±SD) Age (years)<br>% Female/Male<br>Charlson comorbidity index<br>CHA_2DS_2-VASc score<br>HAS-BLED score	**Comparator(s)**<br>*n*=patients<br>mean (±SD) Age (years)<br>% Female/Male<br>Charlson comorbidity index<br>CHA_2DS_2-VASc score<br>HAS-BLED score	**Outcomes**	**Follow-up**	**Impact of Apixaban**<br><br>+ Favours Apixaban<br>- Favours comparator<br>= No difference / uncertain

AF; atrial fibrillation, CRNM; clinically relevant non-major (bleeding), GI; gastrointestinal, ICH; intracerebral haemorrhage, INR; international normalised ratio, NR; not reported, SAH; subarachnoid haemorrhage, SE; systemic embolism.

CHA_2DS_2-VASC; is a stroke risk score for people with AF and includes congestive heart failure, hypertension, age ≥75 (doubled), diabetes, stroke (doubled), vascular disease, age 65 to 74 and sex category (female).

HAS-BLED; is a risk score for major bleeding for patients on anticoagulation to assess risk-benefit in atrial fibrillation care and includes hypertension (uncontrolled, >160mmHg systolic), renal disease, liver disease, stroke history, prior major bleeding, labile INR, age >65, medication use predisposing to bleeding, alcohol use.

Supplement S3.

Table S2. Risk of bias according to the ROBINS-I tool for the included cohort studies.

	**ROBINS-I sub-category**							
**Study**	**Confounding**	**Selection**	**Classification of Interventions**	**Deviation from Intended Interventions**	**Missing Data**	**Measurement of Outcomes**	**Selection of Reported Results**	**Overall**
Abraham 2017	Moderate	Low	Low	Low	Low	Low	Low	**Moderate**
Adeboyeje 2017	Moderate	Low	Low	Low	Low	Low	Low	**Moderate**
Alcusky 2020	Moderate	Low	Low	Low	Low	Low	Low	**Moderate**
Al-Khalili 2016	Moderate	Low	Low	Low	Low	Low	Low	**Moderate**
Amin 2017	Moderate	Low	Low	Moderate	Low	Low	Low	**Moderate**
Amin 2018	Moderate	Low	Low	Low	Low	Low	Moderate	**Moderate**
Amin 2019(1)	Moderate	Low	Low	Low	Low	Low	Low	**Moderate**
Amin 2019(2)	Moderate	Low	Low	Moderate	Low	Low	Low	**Moderate**
Andersson 2018	Moderate	Low	Low	Low	Low	Low	Low	**Moderate**
Bang 2020	Moderate	Low	Low	Moderate	Low	Low	Low	**Moderate**
Bradley 2020	Moderate	Low	Low	Low	Low	Low	Low	**Moderate**
Chan 2018	Moderate	Low	Low	Low	Low	Low	Low	**Moderate**
Coleman 2016	Moderate	Low	Low	Low	Low	Low	Low	**Moderate**
Coleman 2017	Moderate	Low	Low	Low	Moderate	Low	Low	**Moderate**
Coleman 2018	Moderate	Low	Low	Low	Moderate	Low	Low	**Moderate**
Deitelzweig 2016	Moderate	Low	Low	Low	Low	Low	Low	**Moderate**
Deitelzweig 2017	Moderate	Low	Low	Low	Moderate	Low	Moderate	**Moderate**
Durand 2020	Moderate	Low	Low	Low	Low	Low	Low	**Moderate**
Fralick 2020	Moderate	Low	Low	Low	Moderate	Moderate	Low	**Moderate**
Graham 2019	Moderate	Low	Low	Low	Moderate	Moderate	Low	**Moderate**

	ROBINS-I sub-category						
**Study**	**Confounding**	**Selection**	**Classification of Interventions**	**Deviation from Intended Interventions**	**Missing Data**	**Measurement of Outcomes**	**Selection of Reported Results**	**Overall**
Gupta 2019	Moderate	Low	Low	Low	Moderate	Low	Low	**Moderate**
Halvorsen 2017	Moderate	Low	Low	Low	Low	Moderate	Low	**Moderate**
Hernandez 2017	Moderate	Low	Low	Low	Moderate	Moderate	Low	**Moderate**
Hohnloser 2017	Moderate	Low	Low	Low	Low	Moderate	Low	**Moderate**
Hohnloser 2018	Moderate	Low	Low	Low	Low	Moderate	Low	**Moderate**
Huybrechts 2020	Moderate	Moderate	Low	Low	Low	Low	Low	**Moderate**
Jansson 2020	Moderate	Low	Low	Low	Low	Low	Low	**Moderate**
Keshishian 2016	Moderate	Low	Low	Low	Low	Low	Low	**Moderate**
Kjerpeseth 2019	Moderate	Moderate	Low	Low	Low	Moderate	Low	**Moderate**
Kohsaka 2017	Moderate	Low	Low	Low	Low	Low	Low	**Moderate**
Kohsaka 2018	Moderate	Low	Low	Low	Low	Low	Low	**Moderate**
Lamberts 2017	Moderate	Low	Low	Moderate	Low	Low	Low	**Moderate**
Larsen 2016	Moderate	Low	Low	Low	Low	Low	Low	**Moderate**
Lee 2019	Moderate	Low	Low	Low	Low	Low	Low	**Moderate**
Li 2017	Moderate	Low	Low	Moderate	Low	Low	Low	**Moderate**
Lin 2017	Moderate	Low	Low	Low	Low	Low	Low	**Moderate**
Lip 2016(1)	Moderate	Low	Low	Low	Low	Low	Low	**Moderate**
Lip 2016(2)	Moderate	Low	Low	Low	Low	Low	Low	**Moderate**
Lip 2017	Moderate	Low	Low	Low	Low	Low	Low	**Moderate**
Lip 2018	Moderate	Low	Low	Low	Low	Low	Low	**Moderate**
Lip 2020	Moderate	Low	Low	Low	Low	Low	Low	**Moderate**
Martinez 2018	Moderate	Low	Low	Low	Low	Low	Low	**Moderate**
Mentias 2020	Moderate	Low	Low	Low	Low	Low	Low	**Moderate**
Mitsuntisuk 20201	Moderate	Moderate	Low	Low	Low	Low	Low	**Moderate**
Mueller 2019	Moderate	Low	Low	Low	Low	Low	Moderate	**Moderate**

	ROBINS-I sub-category							
**Study**	**Confounding**	**Selection**	**Classification of Interventions**	**Deviation from Intended Interventions**	**Missing Data**	**Measurement of Outcomes**	**Selection of Reported Results**	**Overall**
Nielson 2017	Moderate	Low	Moderate	Low	Low	Low	Low	**Moderate**
Noseworthy 2016	Moderate	Low	Low	Low	Low	Low	Low	**Moderate**
Noseworthy 2017	Serious	Low	Low	Low	Low	Low	Low	**Serious**
Perrault 2021	Moderate	Low	Low	Low	Low	Low	Low	**Moderate**
Ramagopalan 2018	Moderate	Low	Low	Low	Low	Low	Low	**Moderate**
Ramagopalan 2019	Moderate	Low	Low	Low	Low	Low	Low	**Moderate**
Ray 2021	Moderate	Low	Low	Low	Low	Low	Low	**Moderate**
Rodriguezbernal 2021	Moderate	Low	Low	Low	Low	Low	Low	**Moderate**
Rutherford 2020	Moderate	Low	Low	Low	Low	Low	Low	**Moderate**
Själander 2018	Moderate	Low	Low	Low	Low	Low	Low	**Moderate**
Staerk 2017	Moderate	Low	Low	Low	Low	Low	Low	**Moderate**
Staerk 2018	Moderate	Low	Low	Low	Low	Low	Low	**Moderate**
Tepper 2018	Moderate	Low	Low	Low	Low	Low	Low	**Moderate**
Tiew 2020	Moderate	Low	Low	Low	Low	Low	Low	**Moderate**
Van Ganse 2020	Moderate	Low	Low	Low	Moderate	Moderate	Low	**Moderate**
Villanes 2019	Moderate	Low	Low	Low	Moderate	Moderate	Low	**Moderate**
Vinogradova 2018	Moderate	Low	Low	Low	Low	Low	Low	**Moderate**
Wanat 2019	Moderate	Low	Low	Low	Moderate	Low	Low	**Moderate**
Yang 2020	Moderate	Low	Low	Low	Low	Low	Low	**Moderate**
Yao 2016	Moderate	Low	Low	Low	Moderate	Low	Low	**Moderate**

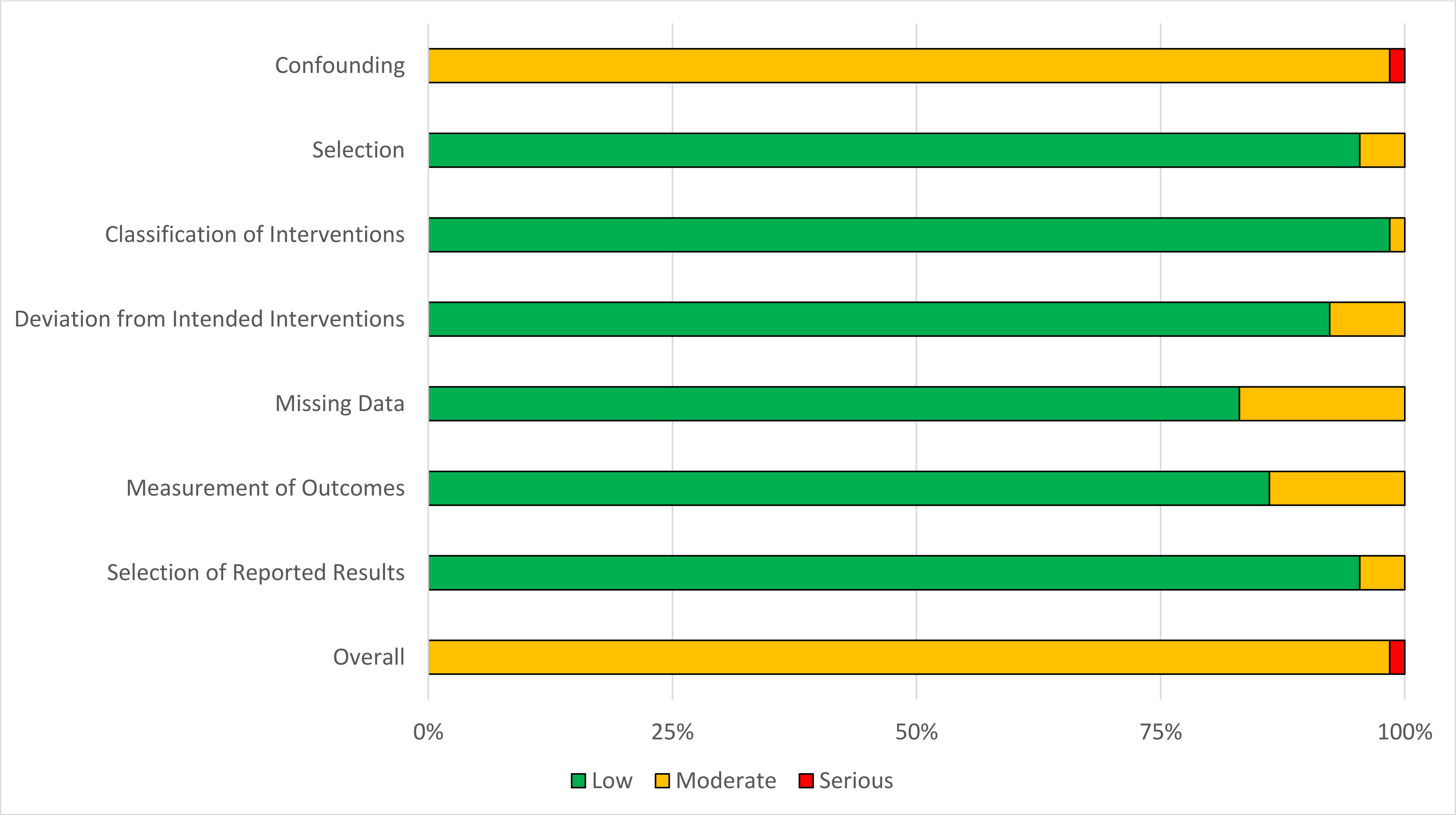

Figure S1. Graphical representation of the Risk of bias according to the ROBINS-I tool for the included cohort studies.

Supplement S4. Sub-group meta-analyses

A

Study or Subgroup	Apixaban Events	Apixaban Total	Comparator Events	Comparator Total	Weight	Risk Ratio M-H, Random, 95% CI
**6.1.1 North America**						
Graham, 2019	190	72921	814	183003	6.1%	0.59 [0.50, 0.69]
Li, 2017	394	38470	609	38470	6.2%	0.65 [0.57, 0.73]
Lip, 2017	10	1470	57	7674	3.4%	0.92 [0.47, 1.79]
Lip, 2018	697	100977	1299	100977	6.3%	0.54 [0.49, 0.59]
Martinez, 2018	24	1392	29	1392	4.1%	0.83 [0.48, 1.41]
Wanat, 2019	121	10189	167	10189	5.8%	0.72 [0.57, 0.91]
**Subtotal (95% CI)**		**225419**		**341705**	**31.9%**	**0.62 [0.55, 0.71]**
Total events	1436		2975			
Heterogeneity: $Tau^2 = 0.01$; $Chi^2 = 12.38$, df = 5 (P = 0.03); $I^2 = 60\%$						
Test for overall effect: Z = 7.45 (P < 0.00001)						
**6.1.2 Asia**						
Bang, 2020	419	10548	413	8648	6.2%	0.83 [0.73, 0.95]
Chan, 2018	100	5843	929	19375	5.9%	0.36 [0.29, 0.44]
Kohsaka, 2018	80	11972	111	11972	5.5%	0.72 [0.54, 0.96]
Mitsuntisuk, 2021	14	405	47	605	3.9%	0.44 [0.25, 0.80]
Nielsen, 2017	236	4400	1686	38893	6.2%	1.24 [1.08, 1.41]
Tiew, 2020	12	98	3	157	1.6%	6.41 [1.85, 22.14]
**Subtotal (95% CI)**		**33266**		**79650**	**29.3%**	**0.80 [0.50, 1.27]**
Total events	861		3189			
Heterogeneity: $Tau^2 = 0.28$; $Chi^2 = 119.26$, df = 5 (P < 0.00001); $I^2 = 96\%$						
Test for overall effect: Z = 0.94 (P = 0.35)						
**6.1.3 Europe**						
Hohnloser, 2018	226	10117	597	23823	6.1%	0.89 [0.77, 1.04]
Kjerpeseth, 2019	189	10550	124	6435	5.8%	0.93 [0.74, 1.16]
Larsen, 2016	210	6349	1004	35436	6.1%	1.17 [1.01, 1.35]
Lip, 2017	10	1470	57	7674	3.4%	0.92 [0.47, 1.79]
Nielsen, 2017	236	4400	1686	38893	6.2%	1.24 [1.08, 1.41]
Ramagopalan, 2019	44	2160	80	2160	5.1%	0.55 [0.38, 0.79]
Staerk, 2017	171	6899	419	18094	6.0%	1.07 [0.90, 1.28]
**Subtotal (95% CI)**		**41945**		**132515**	**38.8%**	**0.98 [0.84, 1.15]**
Total events	1086		3967			
Heterogeneity: $Tau^2 = 0.03$; $Chi^2 = 25.99$, df = 6 (P = 0.0002); $I^2 = 77\%$						
Test for overall effect: Z = 0.21 (P = 0.84)						
**Total (95% CI)**		**300630**		**553870**	**100.0%**	**0.80 [0.67, 0.96]**
Total events	3383		10131			
Heterogeneity: $Tau^2 = 0.14$; $Chi^2 = 304.17$, df = 18 (P < 0.00001); $I^2 = 94\%$						
Test for overall effect: Z = 2.42 (P = 0.02)						
Test for subgroup differences: $Chi^2 = 19.67$, df = 2 (P < 0.0001), $I^2 = 89.8\%$						

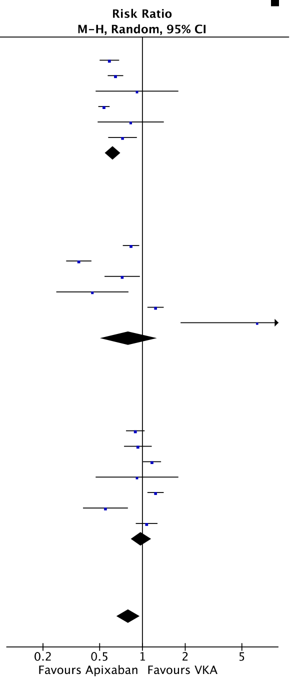

B

Study or Subgroup	Apixaban Events	Apixaban Total	Comparator Events	Comparator Total	Weight	Risk Ratio M-H, Random, 95% CI
**7.1.1 North America**						
Alcusky, 2020	554	2881	645	2881	9.8%	0.86 [0.78, 0.95]
Graham, 2019	456	72921	2234	183003	9.8%	0.51 [0.46, 0.57]
**Subtotal (95% CI)**		**75802**		**185884**	**19.7%**	**0.66 [0.40, 1.11]**
Total events	1010		2879			
Heterogeneity: $Tau^2 = 0.14$; $Chi^2 = 52.57$, df = 1 (P < 0.00001); $I^2 = 98\%$						
Test for overall effect: Z = 1.56 (P = 0.12)						
**7.1.2 Asia**						
Chan, 2018	319	5843	2588	19375	9.8%	0.41 [0.37, 0.46]
Mitsuntisuk, 2021	3	405	4	605	3.6%	1.12 [0.25, 4.98]
**Subtotal (95% CI)**		**6248**		**19980**	**13.4%**	**0.51 [0.23, 1.15]**
Total events	322		2592			
Heterogeneity: $Tau^2 = 0.22$; $Chi^2 = 1.75$, df = 1 (P = 0.19); $I^2 = 43\%$						
Test for overall effect: Z = 1.63 (P = 0.10)						
**7.1.3 Europe**						
Hohnloser, 2018	804	10117	1595	23823	9.9%	1.19 [1.09, 1.29]
Larsen, 2016	232	6349	2652	35436	9.8%	0.49 [0.43, 0.56]
Lip, 2017	19	1470	249	7674	8.5%	0.40 [0.25, 0.63]
Nielsen, 2017	1040	4400	5366	38893	9.9%	1.71 [1.62, 1.82]
Rodríguez-Bernal, 2018	166	2259	4539	32476	9.7%	0.53 [0.45, 0.61]
Vinogradova, 2018, (CPRD Database)	56	1402	780	16664	9.4%	0.85 [0.65, 1.11]
Vinogradova, 2018, (Qresearch Database)	472	9199	3182	53921	9.8%	0.87 [0.79, 0.96]
**Subtotal (95% CI)**		**35196**		**208887**	**66.9%**	**0.78 [0.52, 1.16]**
Total events	2789		18363			
Heterogeneity: $Tau^2 = 0.28$; $Chi^2 = 540.95$, df = 6 (P < 0.00001); $I^2 = 99\%$						
Test for overall effect: Z = 1.22 (P = 0.22)						
**Total (95% CI)**		**117246**		**414751**	**100.0%**	**0.72 [0.50, 1.02]**
Total events	4121		23834			
Heterogeneity: $Tau^2 = 0.33$; $Chi^2 = 1017.02$, df = 10 (P < 0.00001); $I^2 = 99\%$						
Test for overall effect: Z = 1.84 (P = 0.07)						
Test for subgroup differences: $Chi^2 = 0.90$, df = 2 (P = 0.64), $I^2 = 0\%$						

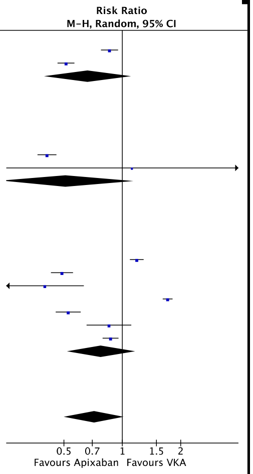

C

Study or Subgroup	Apixaban Events	Apixaban Total	Comparator Events	Comparator Total	Weight	Risk Ratio M-H, Random, 95% CI
**8.1.1 North America**						
Adeboyeje, 2017	245	3689	1729	23431	6.3%	0.90 [0.79, 1.02]
Li, 2017	753	38470	1303	38470	6.6%	0.58 [0.53, 0.63]
Lip, 2018	1902	100977	3770	100977	6.7%	0.50 [0.48, 0.53]
Martinez, 2018	44	1392	58	1392	3.8%	0.76 [0.52, 1.11]
Wanat, 2019	600	10189	887	10189	6.5%	0.68 [0.61, 0.75]
**Subtotal (95% CI)**		**154717**		**174459**	**29.8%**	**0.66 [0.54, 0.81]**
Total events	3544		7747			
Heterogeneity: $Tau^2 = 0.05$; $Chi^2 = 80.57$, df = 4 (P < 0.00001); $I^2 = 95\%$						
Test for overall effect: Z = 3.93 (P < 0.0001)						
**8.1.2 Asia**						
Bang, 2020	432	10548	466	8648	6.3%	0.76 [0.67, 0.86]
Chan, 2018	67	5843	855	19375	5.1%	0.26 [0.20, 0.33]
Kohsaka, 2018	99	11972	134	11972	5.0%	0.74 [0.57, 0.96]
Lee, 2019	290	22177	569	25420	6.2%	0.58 [0.51, 0.67]
Mitsuntisuk, 2021	26	405	96	605	3.5%	0.40 [0.27, 0.61]
Tiew, 2020	4	98	8	157	0.8%	0.80 [0.25, 2.59]
**Subtotal (95% CI)**		**51043**		**66177**	**26.8%**	**0.53 [0.38, 0.75]**
Total events	918		2128			
Heterogeneity: $Tau^2 = 0.15$; $Chi^2 = 65.77$, df = 5 (P < 0.00001); $I^2 = 92\%$						
Test for overall effect: Z = 3.56 (P = 0.0004)						
**8.1.3 Europe**						
Hohnloser, 2018	167	10117	692	23823	5.9%	0.57 [0.48, 0.67]
Kjerpeseth, 2019	446	10550	490	6435	6.3%	0.56 [0.49, 0.63]
Lamberts, 2017	252	7963	1128	24230	6.2%	0.68 [0.59, 0.78]
Larsen, 2016	90	6349	725	35436	5.4%	0.69 [0.56, 0.86]
Nielsen, 2017	160	4400	2136	38893	6.0%	0.66 [0.57, 0.78]
Ramagopalan, 2019	52	2160	100	2160	4.3%	0.52 [0.37, 0.72]
Vinogradova, 2018, (CPRD Database)	22	1402	515	16664	3.4%	0.51 [0.33, 0.78]
Vinogradova, 2018, (Qresearch Database)	119	9199	1813	53921	5.8%	0.38 [0.32, 0.46]
**Subtotal (95% CI)**		**52140**		**201562**	**43.3%**	**0.57 [0.50, 0.65]**
Total events	1308		7599			
Heterogeneity: $Tau^2 = 0.03$; $Chi^2 = 31.24$, df = 7 (P < 0.0001); $I^2 = 78\%$						
Test for overall effect: Z = 7.90 (P < 0.00001)						
**Total (95% CI)**		**257900**		**442198**	**100.0%**	**0.58 [0.52, 0.65]**
Total events	5770		17474			
Heterogeneity: $Tau^2 = 0.05$; $Chi^2 = 176.95$, df = 18 (P < 0.00001); $I^2 = 90\%$						
Test for overall effect: Z = 9.51 (P < 0.00001)						
Test for subgroup differences: $Chi^2 = 1.69$, df = 2 (P = 0.43), $I^2 = 0\%$						

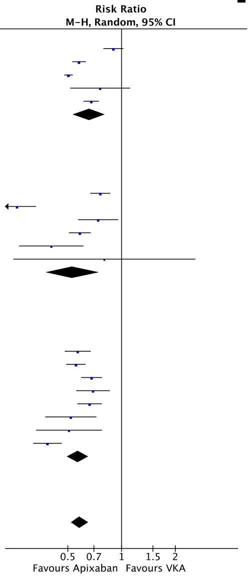

Figure S2. Comparison of apixaban vs VKAs stratified by geographic region (North America, Asia, Europe) for the outcomes stroke/SE (Panel A), mortality (Panel B), and major bleeding (Panel C).

A

Study or Subgroup	Apixaban Events	Apixaban Total	Comparator Events	Comparator Total	Weight	Risk Ratio M-H, Random, 95% CI
**6.4.1 North America**						
Graham, 2019	190	72921	279	86293	12.3%	0.81 [0.67, 0.97]
Lip, 2018	215	37314	333	37314	12.7%	0.65 [0.54, 0.77]
**Subtotal (95% CI)**		**110235**		**123607**	**25.0%**	**0.72 [0.58, 0.89]**
Total events	405		612			
Heterogeneity: $Tau^2 = 0.02$; $Chi^2 = 2.99$, df = 1 (P = 0.08); $I^2 = 67\%$						
Test for overall effect: Z = 2.97 (P = 0.003)						
**6.4.2 Asia**						
Mitsuntisuk, 2021	14	405	25	441	4.0%	0.61 [0.32, 1.16]
Tiew, 2020	12	98	0	30	0.3%	7.83 [0.48, 128.45]
**Subtotal (95% CI)**		**503**		**471**	**4.2%**	**1.55 [0.12, 19.70]**
Total events	26		25			
Heterogeneity: $Tau^2 = 2.54$; $Chi^2 = 3.37$, df = 1 (P = 0.07); $I^2 = 70\%$						
Test for overall effect: Z = 0.34 (P = 0.73)						
**6.4.3 Europe**						
Hohnloser, 2018	226	10117	104	5122	11.2%	1.10 [0.87, 1.38]
Jansson, 2020	115	11493	91	6453	10.0%	0.71 [0.54, 0.93]
Nielsen, 2017	236	4400	535	8875	13.2%	0.89 [0.77, 1.03]
Rutherford, 2020	335	10413	360	10413	13.3%	0.93 [0.80, 1.08]
Staerk, 2017	171	6899	336	12613	12.4%	0.93 [0.78, 1.12]
Staerk, 2018	148	7203	102	7078	10.6%	1.43 [1.11, 1.83]
**Subtotal (95% CI)**		**50525**		**50554**	**70.7%**	**0.97 [0.84, 1.12]**
Total events	1231		1528			
Heterogeneity: $Tau^2 = 0.02$; $Chi^2 = 16.93$, df = 5 (P = 0.005); $I^2 = 70\%$						
Test for overall effect: Z = 0.40 (P = 0.69)						
**Total (95% CI)**		**161263**		**174632**	**100.0%**	**0.89 [0.77, 1.03]**
Total events	1662		2165			
Heterogeneity: $Tau^2 = 0.04$; $Chi^2 = 38.36$, df = 9 (P < 0.0001); $I^2 = 77\%$						
Test for overall effect: Z = 1.53 (P = 0.13)						
Test for subgroup differences: $Chi^2 = 5.20$, df = 2 (P = 0.07), $I^2 = 61.6\%$						

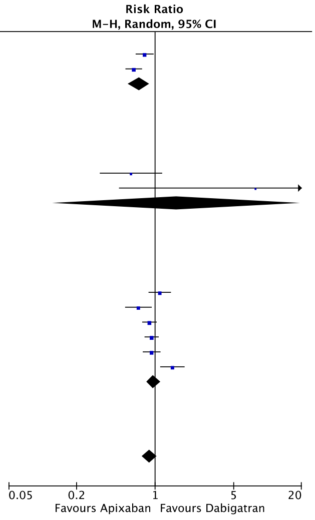

B

Study or Subgroup	Apixaban Events	Apixaban Total	Comparator Events	Comparator Total	Weight	Risk Ratio M-H, Random, 95% CI
**7.2.1 North America**						
Graham, 2019	456	72921	677	86293	13.4%	0.80 [0.71, 0.90]
**Subtotal (95% CI)**		**72921**		**86293**	**13.4%**	**0.80 [0.71, 0.90]**
Total events	456		677			
Heterogeneity: Not applicable						
Test for overall effect: Z = 3.76 (P = 0.0002)						
**7.2.2 Asia**						
**Subtotal (95% CI)**		**0**		**0**		**Not estimable**
Total events	0		0			
Heterogeneity: Not applicable						
Test for overall effect: Not applicable						
**7.2.3 Europe**						
Al-Khalili, 2016	1	251	1	233	0.5%	0.93 [0.06, 14.76]
Hohnloser, 2018	804	10117	253	5122	13.2%	1.61 [1.40, 1.85]
Jansson, 2020	496	11493	246	6453	13.1%	1.13 [0.97, 1.31]
Mueller, 2019	305	6200	61	1112	11.3%	0.90 [0.69, 1.17]
Nielsen, 2017	1040	4400	1873	8875	13.9%	1.12 [1.05, 1.20]
Rodríguez-Bernal, 2018	166	2259	426	3380	12.8%	0.58 [0.49, 0.69]
Vinogradova, 2018, (CPRD Database)	56	1402	38	1003	8.9%	1.05 [0.70, 1.58]
Vinogradova, 2018, (Qresearch Database)	472	9199	212	4534	13.0%	1.10 [0.94, 1.29]
**Subtotal (95% CI)**		**45321**		**30712**	**86.6%**	**1.04 [0.84, 1.28]**
Total events	3340		3110			
Heterogeneity: $Tau^2 = 0.07$; $Chi^2 = 85.00$, df = 7 (P < 0.00001); $I^2 = 92\%$						
Test for overall effect: Z = 0.32 (P = 0.75)						
**Total (95% CI)**		**118242**		**117005**	**100.0%**	**1.00 [0.82, 1.22]**
Total events	3796		3787			
Heterogeneity: $Tau^2 = 0.07$; $Chi^2 = 109.61$, df = 8 (P < 0.00001); $I^2 = 93\%$						
Test for overall effect: Z = 0.00 (P = 1.00)						
Test for subgroup differences: $Chi^2 = 4.39$, df = 1 (P = 0.04), $I^2 = 77.2\%$						

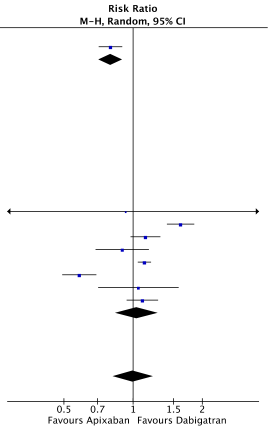

C

Study or Subgroup	Apixaban Events	Apixaban Total	Comparator Events	Comparator Total	Weight	Risk Ratio M-H, Random, 95% CI
**8.2.1 North America**						
Adeboyeje, 2017	62	3689	245	8539	6.5%	0.59 [0.44, 0.77]
Lip, 2018	571	37314	832	37314	9.8%	0.69 [0.62, 0.76]
Tepper, 2018	119	8785	306	20963	7.8%	0.93 [0.75, 1.15]
Villines, 2019	58	4802	77	4802	5.4%	0.75 [0.54, 1.06]
**Subtotal (95% CI)**		**54590**		**71618**	**29.4%**	**0.73 [0.61, 0.88]**
Total events	810		1460			
Heterogeneity: $Tau^2 = 0.02$; $Chi^2 = 8.58$, df = 3 (P = 0.04); $I^2 = 65\%$						
Test for overall effect: Z = 3.39 (P = 0.0007)						
**8.2.2 Asia**						
Lee, 2019	302	22177	263	17745	8.7%	0.92 [0.78, 1.08]
Mitsuntisuk, 2021	26	405	19	441	2.8%	1.49 [0.84, 2.65]
Tiew, 2020	4	98	0	30	0.1%	2.82 [0.16, 50.90]
**Subtotal (95% CI)**		**22680**		**18216**	**11.6%**	**1.07 [0.73, 1.57]**
Total events	332		282			
Heterogeneity: $Tau^2 = 0.05$; $Chi^2 = 3.04$, df = 2 (P = 0.22); $I^2 = 34\%$						
Test for overall effect: Z = 0.37 (P = 0.71)						
**8.2.3 Europe**						
Hohnloser, 2018	167	10117	80	5122	6.7%	1.06 [0.81, 1.38]
Jansson, 2020	224	11493	186	6453	8.1%	0.68 [0.56, 0.82]
Lamberts, 2017	252	7963	695	15413	9.1%	0.70 [0.61, 0.81]
Nielsen, 2017	160	4400	491	8875	8.5%	0.66 [0.55, 0.78]
Rutherford, 2020	845	10413	1169	10413	10.1%	0.72 [0.66, 0.79]
Staerk, 2018	154	7203	114	7078	7.2%	1.33 [1.04, 1.69]
Vinogradova, 2018, (CPRD Database)	22	1402	17	1003	2.5%	0.93 [0.49, 1.73]
Vinogradova, 2018, (Qresearch Database)	119	9199	107	4534	6.8%	0.55 [0.42, 0.71]
**Subtotal (95% CI)**		**62190**		**58891**	**58.9%**	**0.78 [0.66, 0.91]**
Total events	1943		2859			
Heterogeneity: $Tau^2 = 0.04$; $Chi^2 = 38.87$, df = 7 (P < 0.00001); $I^2 = 82\%$						
Test for overall effect: Z = 3.13 (P = 0.002)						
**Total (95% CI)**		**139460**		**148725**	**100.0%**	**0.79 [0.70, 0.88]**
Total events	3085		4601			
Heterogeneity: $Tau^2 = 0.03$; $Chi^2 = 61.04$, df = 14 (P < 0.00001); $I^2 = 77\%$						
Test for overall effect: Z = 4.17 (P < 0.0001)						
Test for subgroup differences: $Chi^2 = 3.22$, df = 2 (P = 0.20), $I^2 = 37.9\%$						

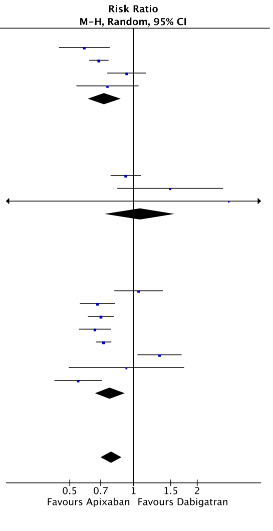

Figure S3. Comparison of apixaban vs dabigatran stratified by geographic region (North America, Asia, Europe) for the outcomes stroke/SE (Panel A), mortality (Panel B), and major bleeding (Panel C).

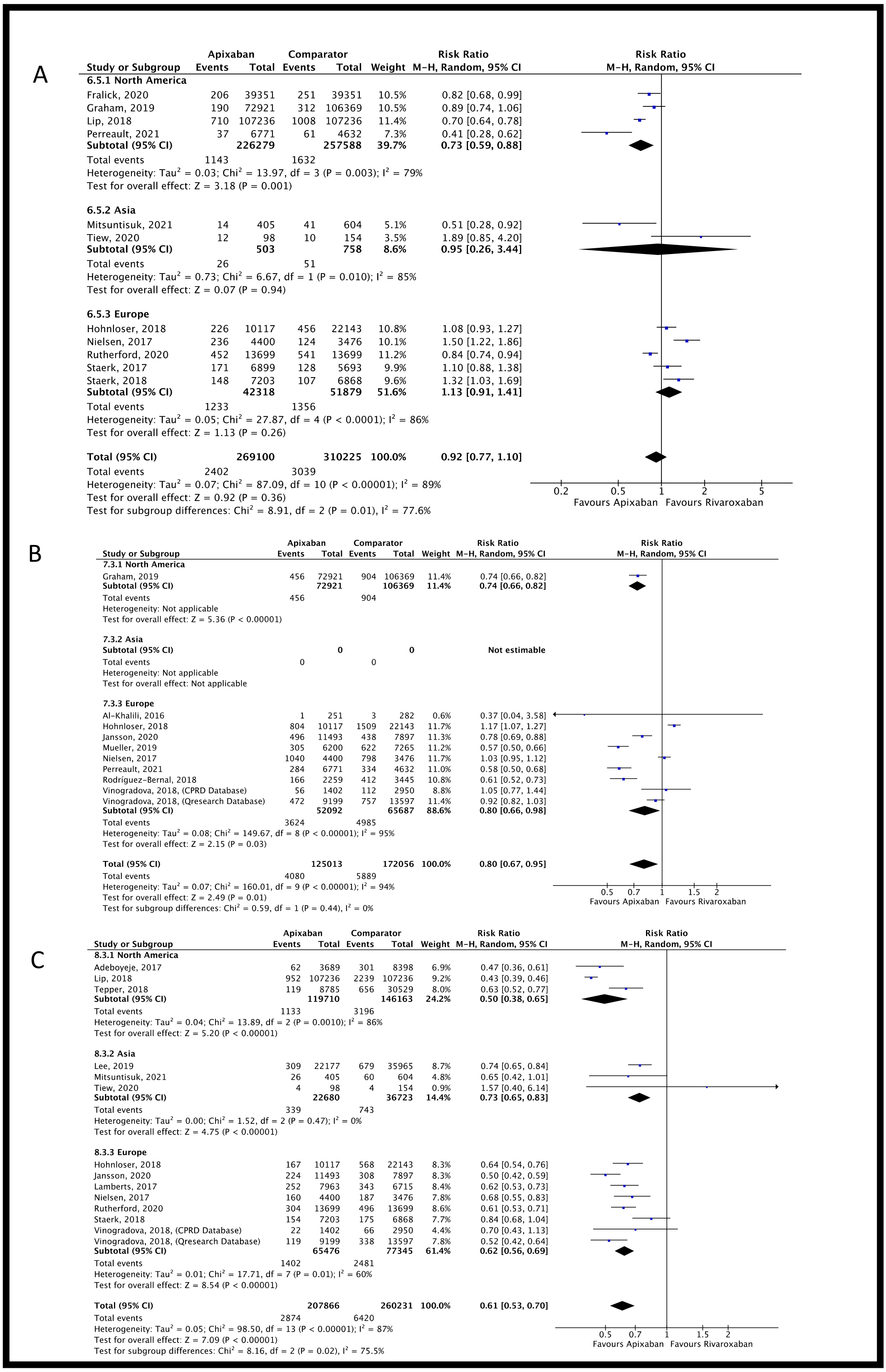

Figure S4. Comparison of apixaban vs rivaroxaban stratified by geographic region (North America, Asia, Europe) for the outcomes stroke/SE (Panel A), mortality (Panel B), and major bleeding (Panel C).
